# Discovery of novel community-relevant small proteins in a simplified human intestinal microbiome

**DOI:** 10.1186/s40168-020-00981-z

**Published:** 2021-02-23

**Authors:** Hannes Petruschke, Christian Schori, Sebastian Canzler, Sarah Riesbeck, Anja Poehlein, Rolf Daniel, Daniel Frei, Tina Segessemann, Johannes Zimmerman, Georgios Marinos, Christoph Kaleta, Nico Jehmlich, Christian H. Ahrens, Martin von Bergen

**Affiliations:** 1 Department of Molecular Systems Biology, Helmholtz-Centre for Environmental Research – UFZ GmbH, Leipzig, Germany; 2 Agroscope, Molecular Diagnostics, Genomics & Bioinformatics and SIB Swiss Institute of Bioinformatics, Wädenswil, Switzerland; 3 Institute of Microbiology and Genetics, Department of Genomic and Applied Microbiology, Georg-August University of Göttingen, Göttingen, Germany; 4 Research Group Medical Systems Biology, Institute for Experimental Medicine, Christian-Albrechts-University Kiel, Kiel, Germany; 5 Institute of Biochemistry, Faculty of Biosciences, Pharmacy and Psychology, University of Leipzig, Leipzig, Germany

**Keywords:** Small proteins (sProteins), SIHUMIx, Human gut microbiome, Proteogenomics, iPtgxDB, Metatranscriptomics, Metaproteomics, Metabolic modelling

## Abstract

**Background:**

The intestinal microbiota plays a crucial role in protecting the host from pathogenic microbes, modulating immunity and regulating metabolic processes. We studied the simplified human intestinal microbiota (SIHUMIx) consisting of eight bacterial species with a particular focus on the discovery of novel small proteins with less than 100 amino acids (= sProteins), some of which may contribute to shape the simplified human intestinal microbiota. Although sProteins carry out a wide range of important functions, they are still often missed in genome annotations, and little is known about their structure and function in individual microbes and especially in microbial communities.

**Results:**

We created a multi-species integrated proteogenomics search database (iPtgxDB) to enable a comprehensive identification of novel sProteins. Six of the eight SIHUMIx species, for which no complete genomes were available, were sequenced and de novo assembled. Several proteomics approaches including two earlier optimized sProtein enrichment strategies were applied to specifically increase the chances for novel sProtein discovery. The search of tandem mass spectrometry (MS/MS) data against the multi-species iPtgxDB enabled the identification of 31 novel sProteins, of which the expression of 30 was supported by metatranscriptomics data. Using synthetic peptides, we were able to validate the expression of 25 novel sProteins. The comparison of sProtein expression in each single strain versus a multi-species community cultivation showed that six of these sProteins were only identified in the SIHUMIx community indicating a potentially important role of sProteins in the organization of microbial communities. Two of these novel sProteins have a potential antimicrobial function. Metabolic modelling revealed that a third sProtein is located in a genomic region encoding several enzymes relevant for the community metabolism within SIHUMIx.

**Conclusions:**

We outline an integrated experimental and bioinformatics workflow for the discovery of novel sProteins in a simplified intestinal model system that can be generically applied to other microbial communities. The further analysis of novel sProteins uniquely expressed in the SIHUMIx multi-species community is expected to enable new insights into the role of sProteins on the functionality of bacterial communities such as those of the human intestinal tract.

Video abstract

**Supplementary Information:**

The online version contains supplementary material available at 10.1186/s40168-020-00981-z.

## Background

The human intestinal microbiota harbors a great potential of functions and microbial interactions. It has a central role in regulating metabolic processes, modulating immunity and protecting the host from pathogenic microbes [1, 2]. Disturbances in the microbial homeostasis can lead to dysbiosis which is associated with various diseases (reviewed in [3, 4]). Given the immense complexity of the intestinal microbiota, however, it is still a challenge to study microbial interactions. Furthermore, it is well-known that the growth and physiology of bacteria in multi-species communities differ from that of individual strains as a consequence of nutrient competition and space constraints [5, 6]. The extended simplified human intestinal microbiota (SIHUMIx) consists of eight common bacterial members of the human intestine and was initially established in a rat model [7] and later as a stable microbial community in continuous flow bioreactors [8, 9]. The reduced complexity compared to the intestinal microbiome allows researchers to use the in vitro model system for the analysis of metabolic output, interdependences, and interactions within SIHUMIx under controlled conditions.

For the analysis of bacterial communities, meta-omics techniques have been developed in the last two decades and metaproteomics allows direct insights into community functions and has been used in several studies of the intestinal microbiota [10–12]. However, this approach depends on protein search databases and is thus limited to the identification of products of previously annotated genes. Within a proteome, small proteins (sProteins) with a size of ≤ 100 amino acids (aa) have been overlooked for a long time, mainly due to challenges in the correct genome annotation of small open reading frames (ORFs) [13, 14], more specifically in differentiating truly coding sProteins from spurious ORFs [15]. Furthermore, the experimental identification of the corresponding gene products poses additional technical challenges [16]. Nevertheless, the interest in sProteins in prokaryotes and eukaryotes has been steadily rising, which can be attributed to the fact that sProteins have been shown to carry out various important functions. In prokaryotes, for example, they play a role in cell division (MciZ, SidA, Blr), transport regulation (SgrT, KdpF), stabilization of membrane-bound enzymes (CydX, PmrR), signal transduction (MgrB) [14], and multi-resistance [17]. Recently, in a large-scale analysis of human microbiomes, Sberro and colleagues predicted about four thousand new sProteins, many of which with previously unknown functions. Their study suggested that sProteins are highly abundant in the intestinal microbiome and perform diverse functions which have not been previously reported. Interestingly, more than 1000 protein families were predicted to either contain transmembrane helices and/or be secreted, suggesting a role in interspecies communication that may help to shape microbial communities [18]. The challenge of such sProtein predictions lies in their experimental validation, since experimental detection methods have not been sufficiently well established. Therefore, it is important to improve the enrichment and comprehensive identification of sProteins in multi-species microbial communities.

In a recent comparison of protocols for LC-MS/MS-based sProtein analysis, including single-pot, solid-phase-enhanced sample preparation (SP3), filter-aided sample preparation (FASP), in-gel- and in-solution proteolytic cleavage, GelFree, and C8-cartridge enrichment, we demonstrated that C8-cartridge and GelFree enrichment significantly increased the number of UniProt annotated sProteins that could be identified in the SIHUMIx model system compared to standard proteomics protocols [19]. However, the fundamental issue that prevents a more comprehensive sProtein discovery remained, namely, that most databases (and the underlying genome annotations) are still incomplete and lack the gene annotations for numerous truly expressed and functional sProteins.

Proteogenomics, a research field at the interface of proteomics and genomics, has the potential to identify expressed unannotated sProteins and thereby overcome the problem of missing sProteins in current genome annotations [20]. By integrating multiple reference genome annotation sources, ab initio gene predictions and all potential in silico ORFs into a single proteogenomics search database (iPtgxDB), numerous novel sProteins, new start sites, and expressed pseudogenes could be directly identified in *Bartonella henselae* based on MS/MS evidence [21]. Subsequently, the general applicability of this concept to other prokaryotes could be established [22, 23].

In this study, we extended the previously developed iPtgxDB approach towards multi-species application to enable the identification of novel sProteins from the SIHUMIx gut microbial community model. The novel sProteins were screened for expression evidence at the RNA level using metatranscriptomics and further validated by comparing the fragmentation pattern of spectra from experimentally identified peptides with those from synthetic peptides. The integrated experimental and bioinformatics workflow for the discovery of novel sProteins can thus be generally applied to other microbial communities.

Novel sProteins uniquely expressed in the communities are expected to provide new insights into important interspecies interactions.

## Material and methods

### Bacteria cultivation

#### Strains of the SIHUMIx community

The SIHUMIx community is composed of eight individual bacterial species, namely *Anaerostipes caccae* (DSMZ 14662), *Bacteroides thetaiotaomicron* (DSMZ 2079), *Bifidobacterium longum* (NCC 2705), *Blautia producta* (DSMZ 2950), *Clostridium butyricum* (DSMZ 10702), *Clostridium ramosum* (DSMZ 1402), *Escherichia coli K-12* (MG1655) and *Lactobacillus plantarum* (DSMZ 20174) [7].

#### Single-strain cultivation

All microbial strains were cultivated separately in brain heart infusion (BHI) medium under anaerobic conditions at 37 °C and continuous shaking at 175 rpm (Supplement Table 1). Single strains of the SIHUMIx community were cultivated both for genomic DNA (gDNA) extraction and sProtein analysis. *A. caccae*, *B. thetaiotaomicron*, *B. longum*, *B. producta*, *C. butyricum*, *C. ramosum* and *L. plantarum* were cultivated as single strains for 48 h. Afterwards, 10 mL bacteria cell suspension were centrifuged (3200×*g*; 10 min; 4 °C) and immediately frozen at − 20 °C for gDNA extraction. Selected single microbial strains (*A. caccae*, *B. thetaiotaomicron*, *B. producta*, and *C. ramosum*) were further cultivated in biological triplicates until they achieved an optical density (OD_600_) between 0.7 and 1.1 (exponential growth phase). Afterward, 10 mL bacteria cell suspension of each replicate were centrifuged (3200×*g*; 10 min; 4 °C) and immediately frozen at − 80 °C for protein extraction.

#### Set-up of the in vitro bioreactor system

To discover novel sProteins, the SIHUMIx was cultured in in vitro bioreactors as previously described [8]. Briefly, the eight bacterial species were cultivated individually for 72 h before inoculation of the bioreactor with 1 × 10^9^ bacterial cells per strain (total cell number = 8 × 10^9^ cells in 250 mL medium). The SIHUMIx community was continuously cultivated in complex intestinal medium (Supplement Table 2) and maintained under anaerobic conditions by continuously gassing the bioreactor vessels with nitrogen [8].

### DNA isolation, sequencing and de novo genome assembly

Cell lysis and gDNA extraction were performed with GenElute™ bacterial genomic DNA kit (Sigma Aldrich, USA). In brief, the bacteria cell pellets were resuspended in 500 μL Lysis Solution T. Cell walls of gram-positive bacteria were destroyed by adding lysozyme (25 mg/mL) and incubation for 2 h at 37 °C, 400 rpm. For further breakdown of cell membranes 0.5 g Zirconia beads (0.1 mm) and 3 glass beads (3 mm) were added and 3 cycles of FastPrep (5.5 ms, 1 min, Fisher Scientific GmbH; Germany) were performed. After centrifugation at 13,000*g* for 5 min the supernatant was mixed with 20 μL RNAse A solution and incubated for 2 min at room temperature to remove RNA. All following steps were performed according to the manufacturer’s instructions. DNA concentration was measured using a Qubit® 2.0 Fluorometer (Thermo Fisher Scientific, USA), and gDNA quality was tested using agarose gel electrophoresis (Supplement Figure 1).

Genomic DNAs of six SIHUMIx strains (*A. caccae*, *B. thetaiotaomicron*, *B. producta*, *C. butyricum*, *L. plantarum*, and *C. ramosum*) were sequenced and de novo assembled using third generation long-read sequencing technologies. In brief, gDNA was sequenced using Pacific Biosciences (PacBio) SMRT technology on an RSII device (1 SMRT cell per strain, P6-C4 chemistry) with a prior enrichment step for fragments > 10 kbp by Blue Pippin (Sage Science; USA). For *B. producta* and *C. butyricum*, additional long reads were generated with an Oxford Nanopore Technology (ONT) MinION flow cell.

The ONT library was prepared by a 1D Sequencing kit (SQK-LSK109) on phenol/chloroform extracted gDNA [24] and sequenced on a FLO-MIN-106D (R9.4.1) flow cell. For all six strains, Illumina MiSeq 2 × 300 bp paired end reads were generated from libraries prepared with a Nextera XT DNA Library Preparation kit (Nextera; UK). The de novo assembly was performed with Flye (v.2.4) [25] using length filtered PacBio RSII subreads (> 5 kb) of *A. caccae*, *B. thethataiomicron*, *C. ramosum*, and *L. plantarum* along with their respective estimated genome sizes (3.2, 6.2, 6.2, and 4.2 Mbp) or length filtered ONT subreads (> 8 kbp and > 20 kbp) of *B. producta* and *C. butyricum* with an estimated genome size of 6.1 Mbp and 4.6 Mbp, respectively*.* Next, assemblies were polished by multiple iterations of Quiver from the SMRT Portal (v.2.3.0.140893) using PacBio Reads (> 1 kbp) until single variant level was reached. To correct any remaining small assembly errors, data from 2 × 300 bp paired end Illumina reads were mapped to the assembly using BWA MEM (v.0.7.17) [26] and FreeBayes (v.1.0.0; minimum alternate fraction: 0.5, minimum alternate count: 5) [27] for 2–3 iterative rounds until no further corrections were detected anymore. Manual start-alignment of the assemblies was set to 200 bp upstream of the *dnaA* gene. To verify the circularity and completeness of the de novo assembly, the filtered PacBio subreads were re-mapped to the circular chromosome using graphmap (v.0.5.2) [28]. Structural variations were called using Sniffles (v.1.0.7) [29] and manually inspected in the integrated genome viewer [30]. Quality parameters for assemblies were calculated by QualiMap (v.2.2.1) [31], and final assemblies were searched by BLASTn (v.2.6.0) against the National Center of Biotechnology Information’s (NCBI) non-redundant RefSeq database (downloaded: 30.09.2019). To detect potential plasmids, which could be missed in the Flye assembly due to size selection for long reads, Plasmid SPAdes (v.3.13.0) [32] was used to assemble the 2 × 300 bp Illumina reads. Cluster of orthologous groups (COGs) of completely or partially (≥ 5 nucleotides) missed genes were determined by EggNOG-mapper (v.2) [33].

### Metatranscriptomics

Harvested cells were resuspended in 800 μL RLT buffer (RNeasy Mini Kit, Qiagen) and cell lysis was performed using a laboratory ball mill. Subsequently, 400 μL RLT buffer (RNeasy Mini Kit Qiagen) and 1200 μL 96% [v/v] ethanol were added. For RNA isolation, the RNeasy Mini Kit (Qiagen) was used as recommended by the manufacturer, but instead of RW1 buffer, RWT buffer (Qiagen) was used in order to also isolate RNAs smaller 200 nt. To determine the RNA integrity number (RIN), the isolated RNA was run on an Agilent Bioanalyzer 2100 using an Agilent RNA 6000 Nano Kit (Agilent Technologies, Germany). Remaining genomic DNA was removed by digesting with TURBO DNase (Invitrogen, ThermoFischer Scientific, UK). The Ribo-Zero magnetic kit (Epicentre Biotechnologies, USA) was used to reduce the amount of rRNA-derived sequences. For sequencing, the strand-specific cDNA libraries were constructed with a NEBNext Ultra directional RNA library preparation kit for Illumina (New England BioLabs, Germany). To assess the quality and size of the libraries, samples were run on an Agilent Bioanalyzer 2100 using an Agilent High Sensitivity DNA Kit (Agilent Technologies, Germany). Concentration of the libraries was determined using the Qubit® dsDNA HS Assay Kit as recommended by the manufacturer (Life Technologies GmbH, Germany). Sequencing was performed on a HiSeq4000 instrument (Illumina Inc., USA) using the HiSeq 3000/4000 SR Cluster Kit for cluster generation and the HiSeq 3000/4000 SBS Kit (50 cycles) for sequencing in the single-end mode and running 1 × 50 cycles.

### Proteome analysis of the microbial cultures

We applied five different protein extraction approaches, including two sProtein enrichment methods to improve the detection of novel sProteins: SP3, FASP, in-solution proteolytic cleavage, C8-cartridge enrichment, and GelFree enrichment as previously described [19]. In this study, enrichment with C8-cartridges and GelFree enrichment led to an increased number of small protein identifications in SIHUMIx. However, the identified sProteins differed widely between the two methods. Global proteomics methods such as SP3, FASP, and in-solution cleavage resulted in fewer sProteins identifications overall, but still added sProteins that were not identified by the enrichment methods [19]. Thus, we used enrichment methods and global proteomics methods for an increased chance of novel sProtein detection. Proteolytic cleavage was performed with either trypsin or Asp-N (further details are described in the Supplement information 1).

#### Mass spectrometry

For each LC-MS/MS run, 5 μL of total peptide solution was injected into nanoHPLC (UltiMate 3000 RSLCnano, Dionex, Thermo Fisher Scientific). Peptides were trapped on a C18-reverse phase trapping column (C18 PepMap100, 300 μm × 5 mm, particle size 3 μm, Thermo Fisher Scientific, or μPAC^TM^ Trapping column, Pharmafluidics, Belgium), followed by separation on a C18-reverse phase analytical column (Acclaim PepMap® 100, 75 μm × 25 cm, particle size 3 μm, nanoViper, Thermo Fisher Scientific, or 50 cm μPAC^TM^ column, Pharmafluidics). Mass spectrometric analysis of eluted peptides was performed on a Q Exactive HF mass spectrometer (Thermo Fisher Scientific, USA) coupled with a TriVersa NanoMate (Advion, UK) source in LC chip coupling mode (Supplement information 2).

#### Database construction

To investigate the full coding potential of the SIHUMIx strains, an iPtgxDB was generated for each strain. In brief, genome annotations retrieved from NCBI’s Prokaryotic Genome Annotation Pipeline (PGAP) [34], ab initio gene predictions from Prodigal (v.2.6.3) [35] and ChemGenome (v.2.1; with parameters: method: SwissProt space; length threshold: 70 nt; initiation codons: ATG, CTG, TTG, GTG) [36], and in silico ORFs (> 18 aa) based on a modified six frame translation (also considering the alternative start codons CTG, TTG, and GTG), were hierarchically integrated as described before [21]. For this, the different genome annotations were collapsed into annotation clusters with the same stop codon but different start sites (considering possible longer proteoforms identifiable by trypsin or Asp-N). Then, the iPtgxDBs of each individual SIHUMIx species (for a given protease) were concatenated to represent the full coding potential of the SIHUMIx culture mix. The NCBI PGAP annotations based on our de novo assembled genomes were retrieved between February and May 2019. For *B. longum* and *E. coli*, NCBI RefSeq annotations from August 2016 and October 2018 were used, respectively.

#### Proteomic data analysis

Mass spectrometric data processing was performed using Proteome Discoverer (v.2.2, Thermo Fischer Scientific, USA) with SequestHT search engine. Search settings were set to trypsin (Full), or Asp-N (Full), max. missed cleavage sites 2, precursor mass tolerance 10 ppm, and fragment mass tolerance 0.05 Da. Carbamidomethylation of cysteines was specified as a fixed modification. False discovery rates (FDR) were determined using Percolator [37]. Proteins were considered as identified when at least one unique peptide was found, the overall protein FDR was ≤ 0.01, and a SequestHT Score of ≥ 2 was reached.

In addition, database searches were also performed with MS-GF+ [38] after converting the raw data to mascot generic file (mgf) format with MSConvert (v.3.0.19184, ProteoWizard [39]) and using the following search parameters: precursor mass tolerance, 10 ppm; fragmentation method, HCD; instrument type, Q Exactive; using fully tryptic or Asp-N peptides only; max charge, 5+; max missed cleavages, 2; and carbamidomethylation of cysteine set as fixed modification. The search was performed against a trypsin- or Asp-N-specific iPtgxDB of all SIHUMIx species and the peptide spectrum match (PSM)-level FDR was estimated using a target-decoy strategy. The search results were filtered to ≥ 2 PSMs and < 0.01 FDR at the protein level.

To increase the stringency for novel protein identifications from Proteome Discoverer and MS-GF+, an additional annotation resource-dependent threshold of required PSMs was applied [21, 23]: (Prodigal and ChemG ≥ 3 PSMs; in silico ≥ 4 PSM) as previously recommended [20]. We furthermore assessed the proteotypicity of the identified peptides using an in-house version of the original PeptideClassifier software [40] further extended to support proteogenomics in prokaryotes [21] and considered peptides that unambiguously identify one protein (so-called class 1a peptides). For this study, we also considered 3a peptides, which unambiguously identify one protein sequence that however can be encoded by different gene models (e.g., duplicated genes).

#### Synthetic peptide measurement

To validate the PSMs of identified novel sProteins, synthetic peptides were ordered from Thermo Fisher Scientific, USA. The synthetic peptides were resolved in 1 mL 40% acetonitrile and 1% formic acid and further diluted to 1 ng/μL. MS/MS spectra were generated by direct infusion with a TriVersa NanoMate (Advion, UK) source coupled to a Q Exactive HF mass spectrometer (Thermo Fisher Scientific, further details are described in the Supplement information 3). The matched peptide spectra were compared to synthetic peptide spectra using *NIST MS Search Program* (v.2.0 g; National Institute of Standards and Technology (NIST), USA) with ± 0.01 *m/z* precursor and ± 0.02 *m/z* product ion tolerance. Novel sProteins were considered to be valid if one of their peptides achieved a match score of ≥ 500 and a reverse match score of ≥ 700.

### Metatranscriptome analysis

To map and count the metatranscriptome reads, we constructed a reference metagenome based on the eight SIHUMIx species (six de novo assembled genomes and two RefSeq genomes). The species-specific chromosomes and plasmids were concatenated resulting in a metagenome with nine different chromosomes and four plasmids. The metagenome annotation database was created by combining the six novel genome annotations (see description above) with the two existing ones. Sequencing adapters were trimmed from the retrieved reads using the cutadapt software (v.1.5) [41]; a subsequent quality control was performed using the fastqc program (v.0.11.2) [42] and the fastx-toolkit (v.0.013). Reads were aligned to the metagenome using the hisat2 mapping tool (v.2.1.0) [43] and subsequently sorted by name and genomic location using samtools (v.1.1) [44]. The number of reads that overlap known genes from the reference annotation was counted using the htseq-count program (v.0.6.1) [45]. The workflow was implemented into our universal analysis pipeline (UAP) workflow management tool [46]. The read counts were normalized using the transcripts per million (TPM) approach.

### sProtein sequence conservation

To estimate the degree of conservation of the identified novel sProteins, a BLASTp against the NCBI protein RefSeq database (2020-05-17) for bacteria (taxid:2) was performed with following settings: e-value of ≤ 10^-5^, a minimum sequence identity of 50% and minimum query coverage of 50%. If multiple homologs were identified, the closest relative hit to query strain was reported.

### In silico structural and functional predictions

Novel SIHUMIx sProteins were further analyzed using multiple tools. The physicochemical properties isoelectric point (p*I*), aliphatic index and grand average of hydropathy score (GRAVY score) of the protein sequences were calculated using ProtParam [47]. Prediction of protein localization was performed by Phobius [48], and prediction of potential antimicrobial peptide (AMP) activity was performed with AMP Scanner (v.2; probability score: > 0.5: potential AMP; < 0.5: non-AMP) [49]. Functional domain prediction was performed by ScanProsite (v.2020_02) [50] and a structural modelling of sProtein candidates by Phyre (v.2.0) [51].

### Microbial community modelling

#### Automated reconstruction of metabolic networks

Metabolic networks were reconstructed using gapseq (v.1.0) [52], which infers metabolic pathways, reactions, and transporter based on genomic data. The default settings were used (bitscore cutoff: 200) and initial gap filling ensured that growth of the metabolic models with flux balance analysis was possible given the complex intestinal medium (Supplement Tables 2 and 9). To this end, we derived the molecular composition of the complex intestinal medium by mapping the molecular constituents of the medium to the corresponding in silico representation of the metabolites.

#### Metabolic modelling of individual species and microbial communities

Microbial community modelling was performed as described previously [53]. Briefly, metabolic networks of the individual strains were joined together within a common extracellular compartment. Coupling constraints were added in order to associate reaction fluxes of each species with its corresponding growth rate. For each species, we added an artificial biomass metabolite that was produced by the biomass reaction and exported into the common extracellular environment. Subsequently, we added an artificial community biomass reaction that drained the individual species’ biomass metabolite according to the relative abundance of each species in the community measured experimentally. The inflow of metabolites into the extracellular space was adjusted according to the composition of the complex intestinal medium. For community modelling, the community-level biomass reaction was set as objective with concomitant minimization of total flux (with a coefficient of 10^−6^ in the optimization function). Similarly, single-species growth was modeled using the individual species’ metabolic networks, constraining exchange reaction according to medium composition and optimizing growth rate with concomitant minimization of total flux. For comparison of fluxes between single growth and community growth, all fluxes were scaled by dividing fluxes with the growth rate (i.e., growth rate of the species in single growth or within the community). To study the role of each reaction for community growth, reactions were knocked out by constraining upper and lower bounds to zero and repeating the optimization.

To study the relevance of sProteins for microbial community metabolism, we identified all enzymes within a 15,000-bp window of each sProtein that showed differential abundance in community growth for each species and identified the reactions that they catalyze. Only enzymes that catalyzed reactions that had non-zero flux in either single or community growth were considered.

### Statistical analysis and visualization

All statistical analyses and plots were performed/created in R (v.3.4.0) [54] using ggplot2 [55]. Circular plots were created with Circos [56]. Stacked bar charts were created using GraphPad Prism (v.8.4.1).

### Availability of data and materials

PacBio, ONT, and Illumina data were uploaded to NCBI’s short read archive (SRA) and can be accessed via the following BioProject and sequence accession numbers: PRJNA523317, CP036345 (*A. caccae*); PRJNA531376, CP039126 (*B. producta*); PRJNA523323, CP036346 (*C. ramosum*); PRJNA531377, CP039121, and CP039122 (*L. plantarum*); PRJNA543750, CP040530, and CP040529 (*B. thetaiotaomicron*); PRJNA544389, CP040626, to CP040629 (*C. butyricum*). The iPtgxDBs can be downloaded from https://iptgxdb.expasy.org/. Metatranscriptomics data can be accessed via the following Bioproject: PRJNA655119; proteomics data (both from individual single-strain cultures and the SIHUMIx grown in the bioreactor have been uploaded to PRIDE and can be assessed under PXD020005.

## Results

### Sequencing and de novo genome assembly of SIHUMIx species

**Fig. 1:**
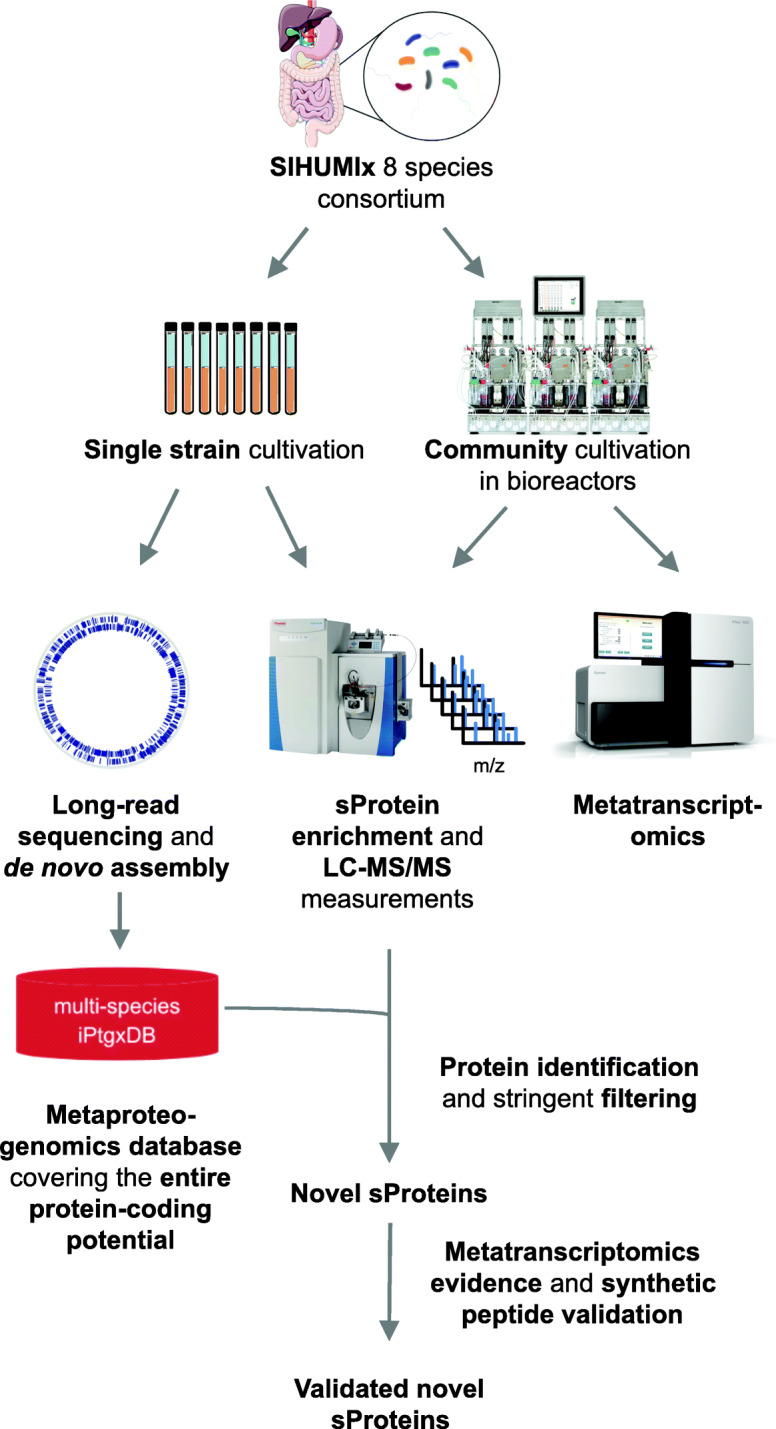
sProtein discovery and validation workflow in a multi-species model: SIHUMIx strains were grown as single cultures for genomic DNA isolation followed by long read sequencing and proteomics measurement. The de novo assembled, complete genomes were used as a basis to create a minimally redundant multispecies-iPtgxDB containing novel sProtein candidates. The SIHUMIx community was cultivated in chemostatic bioreactors and sampled for subsequent proteomics and metatranscriptomics measurement. sProteins of single cultures and the community cultured SIHUMIx strains were enriched and measured by LC-MS/MS. Searching MS/MS spectra against the multi-species iPtgxDB allowed to identify novel sProteins which were further screened for metatranscriptomics expression evidence of the respective genes and validated with synthetic peptides

**Fig. 2:**
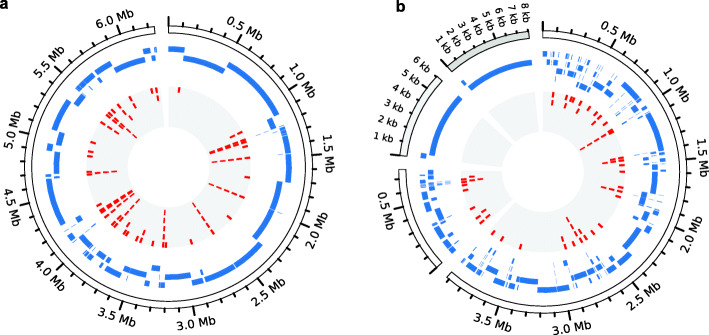
Comparison of complete, long read-based de novo assembled genomes (outer circle showing the size of the bacterial chromosome and plasmids) and the corresponding fragmented, short read-based assemblies. Blue bars denote assembled contigs; missed CDS are shown in red (grey shaded inner circle). **a** *B. producta*. **b** *C. butyricum*

For two of the eight SIHUMIx strains, i.e., *B. longum* and *E. coli*, fully assembled, complete genome sequences were available at NCBI’s RefSeq database. In contrast, for the remaining six species, only fragmented Illumina assemblies (between nine contigs for *Lactobacillus plantarum* to 207 contigs for *Clostridium butyricum*) had been deposited (Supplement Table 3). To create an optimal basis for our subsequent proteogenomics and functional genomics analyses, we first sequenced and de novo assembled the genomes of these six strains using a combination of long reads from the PacBio and ONT platforms and Illumina short reads (Fig. 1). On average, these six complete genomes contained ~ 69 kbp additional sequence information per genome (ranging from 8.3 kb for *A. caccae* to 169.3 kb for *B. producta*) and 94 more genes, which corresponded to roughly 60 protein coding sequences (CDS; between 3 for *C. ramosum* and 198 for *B. producta*) (Table 1), including up to 49 annotated sProteins in a single species (*B. producta*) that otherwise would have been completely or partially missed (Table 1). Among the 364 missed proteins, 259 had an assigned COG category. In total, 92 of these (35%) fell into the COG-category “replication and repair” (including 59 transposases), 24 (9%) into “cell wall/membrane/envelope biogenesis,” and 6 (1%) into “signal transduction mechanisms” which are of particular relevance in the context of multi-species culturing (see Supplement Table 4). These differences are illustrated for *B. producta* and *C. butyricum* (as an example of a strain with additional plasmids) in Fig. 2, and for the remaining strains in Supplement Figure 2.

**Table 1:** Comparison of six de novo assembled SIHUMIx strains to NCBI RefSeq data

Species	Complete de novo genome assembly				Missed in RefSeq entry			
	Genome size	Plasmid(s)	Genes	CDS	Bp	Genes	CDS	CDS ≤ 100aa
***A. caccae***	3,590,716	–	3513	3440	8306	14	10	3
***B. producta***	6,245,307	–	5766	5678	169,296	238	198	49
***B. thetaiotaomicron***	6,271,157	33,036	5027	4940	49,610	61	56	5
***C. butyricum***	3,921,278 (Chr. 1)	6059 (Pl. 1)	4269	4142	115,260	169	74	12
	770,199 (Chr. 2)	8060 (Pl. 2)						
***L. plantarum***	3,242,936	7218	3151	3064	47,954	48	23	11
***C. ramosum***	3,247,604	–	3108	3025	24,277	35	3	0

### Generation of a multi-species iPtgxDB that covers the entire coding potential and its characteristics

To enable the identification of novel sProteins in the SIHUMIx mixture, we relied on a metaproteogenomic approach and an adaptation of our integrated proteogenomics search database approach (iPtgxDB; https://iptgxdb.expasy.org) [21] to this multi-species model. First, individual iPtgxDBs were created for each SIHUMIx genome sequence. By hierarchical integration of reference genome annotations like NCBI RefSeq, ab initio gene prediction algorithms like Prodigal [35] and all in silico ORFs predicted by a modified six-frame translation considering alternative start codons, the iPtgxDBs cover the entire protein coding potential of a genome down to a user-selectable protein size threshold (here 18 aa). To achieve minimal redundancy, only the protein sequence of the annotation source with the highest hierarchy (e.g., RefSeq database) was added in full length. All additional annotations/predictions which imply longer (extensions) or shorter (reductions) protein sequences represent variants of this protein annotation cluster [21], and their sequence up to the first proteolytic cleavage site was added to the iPtgxDB. For this reason, individual iPtgxDBs have to be generated for each protease, in our case for trypsin and Asp-N. The extension of the PeptideCassifier concept [40] to these protein annotation clusters allows to readily identify class 1a peptides (unique to one DB entry), or less frequent class 2a, 3a, or 3b peptides. These are either unique to a subset of sequences of one annotation cluster (2a), unique to a protein sequence that is encoded by different gene models (3a; mainly duplicated genes), or mapping to multiple proteins encoded by different genes (3b; ambiguous identification) [21]. This classification allows to quickly filter unambiguously identified and so far missed sProteins. It can also identify and filter out peptides that arise from proteolytic maturation events of annotated RefSeq proteins that might erroneously imply a novel sProtein. Next, the individual iPtgxDBs were concatenated. Importantly, through this careful hierarchical integration, more than 93% of the proteins included in the iPtgxDB, which covers any potentially missed sProtein, are theoretically MS-identifiable by unique peptides (class 1a). While the iPtgxDB is about 26 times larger compared to RefSeq databases, its search space is still smaller than a six-frame translation of the human genome, which would create a search database 70 times larger than the UniProtKB [20]. The percentage of annotated sProteins in the respective RefSeq genome annotations for the individual SIHUMIx species varied between 4.9 and 12.5% (Table 2). In contrast, the percentage of potentially encoded sProteins increased to almost 90% in the final iPtgxDB (see Table 2 for trypsin, Supplement Table 5 for Asp-N).

**Table 2:** Composition of the multi-species iPtgxDB (trypsin)

Strains	RefSeq proteins	RefSeq sProteins^a^	Extensions to RefSeq sProteins^a^	Additional Prodigal sProteins^a^	Additional ChemGenome sProteins^a^	Additional in silico sProteins^a^	Total iPtgxDB annotation clusters	Total iPtgxDB sProtein annotation clusters^a^
*A. caccae*	3440	295	129	106	2398	78,654	90,548	81,582
*B. longum*	1728	85	55	175	1338	43,506	59,156	45,159
*B. producta*	5682	577	305	306	3749	140,819	165,184	145,756
*B. thetaiotaomicron*	4941	463	274	279	3814	128,815	146,729	133,645
*C. butyricum*	4148	391	131	187	282	68,564	75,453	69,555
*C. ramosum*	3025	281	131	110	218	52,203	56,936	52,943
*E. coli K-12*	4411	551	295	128	3485	106,268	123,548	110,727
*L. plantarum*	3067	384	176	92	1611	72,764	80,137	75,027
Combined iPtgxDB	30,442	3027	1496	1383	16,895	691,593	797,691	714,394

^a^Due to our focus on novel sProtein discovery, we list the respective number of sProteins for a given category

### Identification of novel sProteins

After anaerobic cultivation of the SIHUMIx community, the bacterial cells were harvested and processed using metaproteomics and sProtein enrichment protocols. We used the published data set from Petruschke et al., with the five different protein extraction approaches, (i) SP3, (ii) FASP, (iii) in-solution proteolytic cleavage, (iv) C8 cartridge enrichment, and (v) GelFree enrichment using trypsin as protease [19]. In this study, we performed additional proteomics analysis for three protocols (i) FASP, (ii) C8-cartridge enrichment, and (iii) GelFree enrichment with the protease Asp-N to further increase the detection of novel sProteins that cannot be identified with trypsin [57, 58]. All LC-MS/MS data were searched against the multispecies iPtgxDB (Fig. 1).

**Fig. 3:**
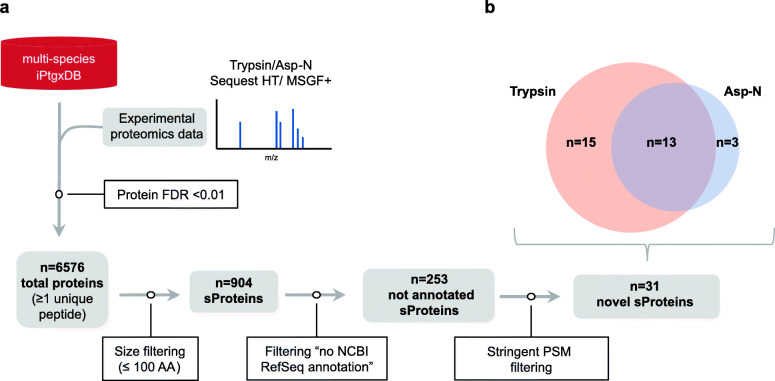
Computational filtering strategy to identify potential novel sProteins. **a** The proteomics data was searched against the multi-species iPtgxDB using SequestHT and MS-GF+ separately. A stringent protein FDR < 0.01 was applied resulting in 6576 total proteins. In total, 904 of these were sProteins of which 253 were not contained in the NCBI RefSeq annotation. An additional prediction resource quality filter (Prodigal und ChemGenome predictions ≥ 3 PSMs; in silico ORF prediction ≥ 4 PSMs) resulted in 31 novel sProteins. **b** Comparison of the number of novel sProteins identified using trypsin and Asp-N as proteolytic enzymes before LC-MS/MS

In total, 6576 proteins were identified, of which 904 (13.7%) were sProteins (Fig. 3a). In total, 253 of these sProteins were not contained in the NCBI RefSeq annotation, and hence represent the entire pool of potential novel sProteins. In line with recommendations to exercise caution when calling novel sProteins [20, 21, 23], we added an additional filtering regimen. To increase the stringency, we applied a prediction resource quality-based filtering step and required at least 3 PSMs for Prodigal and ChemGenome predictions and at least 4 PSMs for in silico ORF predictions. This resulted in a reduction down to 31 novel sProteins (Table 3). Twenty-eight of these novel sProteins were identified by trypsin and 16 by Asp-N. Although the vast majority of the 16 novel sProteins identified by Asp-N were also identified by trypsin (81%), we were still able to identify three novel sProteins uniquely with Asp-N (BP15, BT2, and CR3, Table 3, Supplement Figure 4) that would otherwise have remained undiscovered (Fig. 3b). Moreover, in several cases, the Asp-N search results added additional peptides and PSMs to support identification of a novel sProtein identified with trypsin, e.g., for AC1, BP4, and BP10 (Table 3, Supplement Figure 4). An Asp-N cleavage resulted in slightly longer peptides (average of 17.9 aa vs. 14.1 aa for trypsin). The sProtein BT2 was identified as full protein, as its sequence did not harbor any proteolytic site for Asp-N. In contrast, it contained 13 tryptic cleavage sites, which would produce fragments of maximally 6 aa (avg. length 2.8 aa), i.e., the likely reason why BT2 was not observed in the tryptic digests. A visualization of the PSM distribution for several representative novel sProteins is shown in Supplement Figure 4.

**Table 3:** Overview and identification source of 31 novel sProteins

Abbreviation	Species	Prediction source	Size [aa]	Trypsin		Asp-N	
				Peptides	PSMs	Peptides	PSMs
AC1	*A. caccae*	Prodigal	47	1	5	2	11
AC2	*A. caccae*	Prodigal	49	1	5	0	0
AC3	*A. caccae*	Prodigal	57	3	25	0	0
BL1	*B. longum*	In silico	57	1	5	0	0
BP1	*B. producta*	Prodigal	39	4	12	2	8
BP2	*B. producta*	Prodigal	45	1	5	0	0
BP3	*B. producta*	Prodigal	45	1	19	0	0
BP4	*B. producta*	Prodigal	46	3	12	2	20
BP5	*B. producta*	Prodigal	48	1	11	0	0
BP6	*B. producta*	Prodigal	49	4	25	0	0
BP7	*B. producta*	Prodigal	52	2	9	2	4
BP8	*B. producta*	Prodigal	57	2	12	2	3
BP9	*B. producta*	Prodigal	58	3	23	0	0
BP10	*B. producta*	Prodigal	61	2	50	4	209
BP11	*B. producta*	Prodigal	64	2	6	0	0
BP12	*B. producta*	Prodigal	66	5	34	4	11
BP13	*B. producta*	Prodigal	70	6	547	5	405
BP14	*B. producta*	ChemGenome	81	6	55	2	5
BP15	*B. producta*	ChemGenome	87	0	0	1	4
BT1	*B. thetaiotaomicron*	ChemGenome	32	1	5	0	0
BT2	*B. thetaiotaomicron*	Prodigal	36	0	0	1	16
BT3	*B. thetaiotaomicron*	Prodigal	53	2	3	0	0
BT4	*B. thetaiotaomicron*	Prodigal	55	1	19	1	4
BT5	*B. thetaiotaomicron*	In silico	57	4	30	2	10
BT6	*B. thetaiotaomicron*	Prodigal	57	3	115	5	36
BT7	*B. thetaiotaomicron*	Prodigal	61	1	8	2	19
BT8	*B. thetaiotaomicron*	Prodigal	68	2	35	0	0
CR1	*C. ramosum*	In silico	20	1	4	0	0
CR2	*C. ramosum*	In silico	31	2	18	0	0
CR3	*C. ramosum*	Prodigal	44	0	0	1	3
CR4	*C. ramosum*	Prodigal	58	7	53	3	13

A similarity search revealed that eight of the novel sProteins had no homolog in any other prokaryote (Supplement Table 6). For 12 sProteins, a homologous hypothetical protein in the same species and for 13 sProteins a hypothetical protein in another species was identified. Additionally, the similarity search identified one sProtein as identical to a recently annotated RefSeq protein (peptide chain release factor 2) in *B. thetaiotaomicron* VPI-5482, a strain closely related to our *B. thetaiotaomicron* strain DSM 2079, and whose full genome sequence (NCBI acc: NC_004663.1 (chromosome); NC_004703.1 (plasmid)) had been reported in March 2020. Accordingly, this sProtein was removed from the list of novel sProteins. Furthermore, BP13 exhibited high homology to the N-terminus of a TetR/AcrR family transcriptional regulator in *B. producta*. However, based on a point mutation that introduced an internal stop codon, the encoding gene was annotated as a pseudogene in the NCBI annotation, which we did not integrate here due to our primary focus on novel sProtein discovery. Interestingly, the very high spectral count for this protein (Table 3), which was exclusively observed in the N-terminal 70 aa up to the internal stop codon, provided proteomic proof that BP13 represents a highly expressed proteoform of this pseudogene (Supplement Figure 5). Accordingly, BP13 remained on the list of novel sProteins (Table 3).

**Fig. 4:**
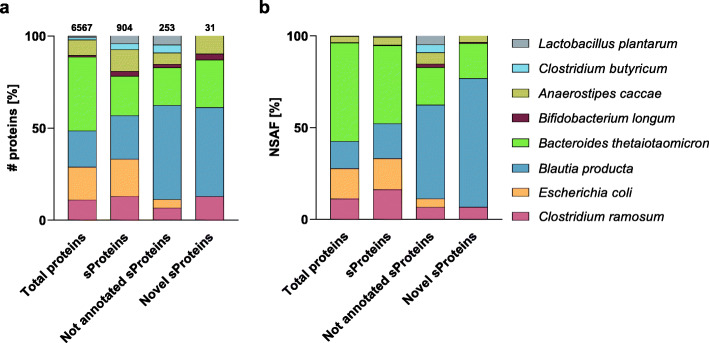
**a** Comparison of the SIHUMIx composition based on the number of proteins identified [%] in four categories with the total protein number for the respective subset shown on top of each bar and **b** their normalized spectral abundance factor (NSAF, [%])

The eight different bacteria species of the SIHUMIx community were not equally represented during cultivation [9]. We first compared the relative number of detected proteins (Fig. 4a). Total proteins, sProteins, and sProteins missed in the respective NCBI RefSeq annotations were identified for all eight SIHUMIx members, with *B. thetaiotaomicron*, *B. producta*, and *E. coli* showing the highest relative number of proteins. After applying the stringent PSM filtering criteria, this number was reduced to 31 novel sProteins. All identified sProteins belong to *B. producta*, *B. thetaiotaomicron*, *C. ramosum*, *A. caccae*, and *B. longum*. The comparison of relative protein abundances based on normalized spectral abundance factor (NSAF) [59] showed a similar result with the highest relative protein abundance observed for *B. thetaiotaomicron* in the case of total proteins and sProteins, and *B. producta* in the case of not annotated sProteins and novel sProteins (Fig. 4b).

**Fig. 5:**
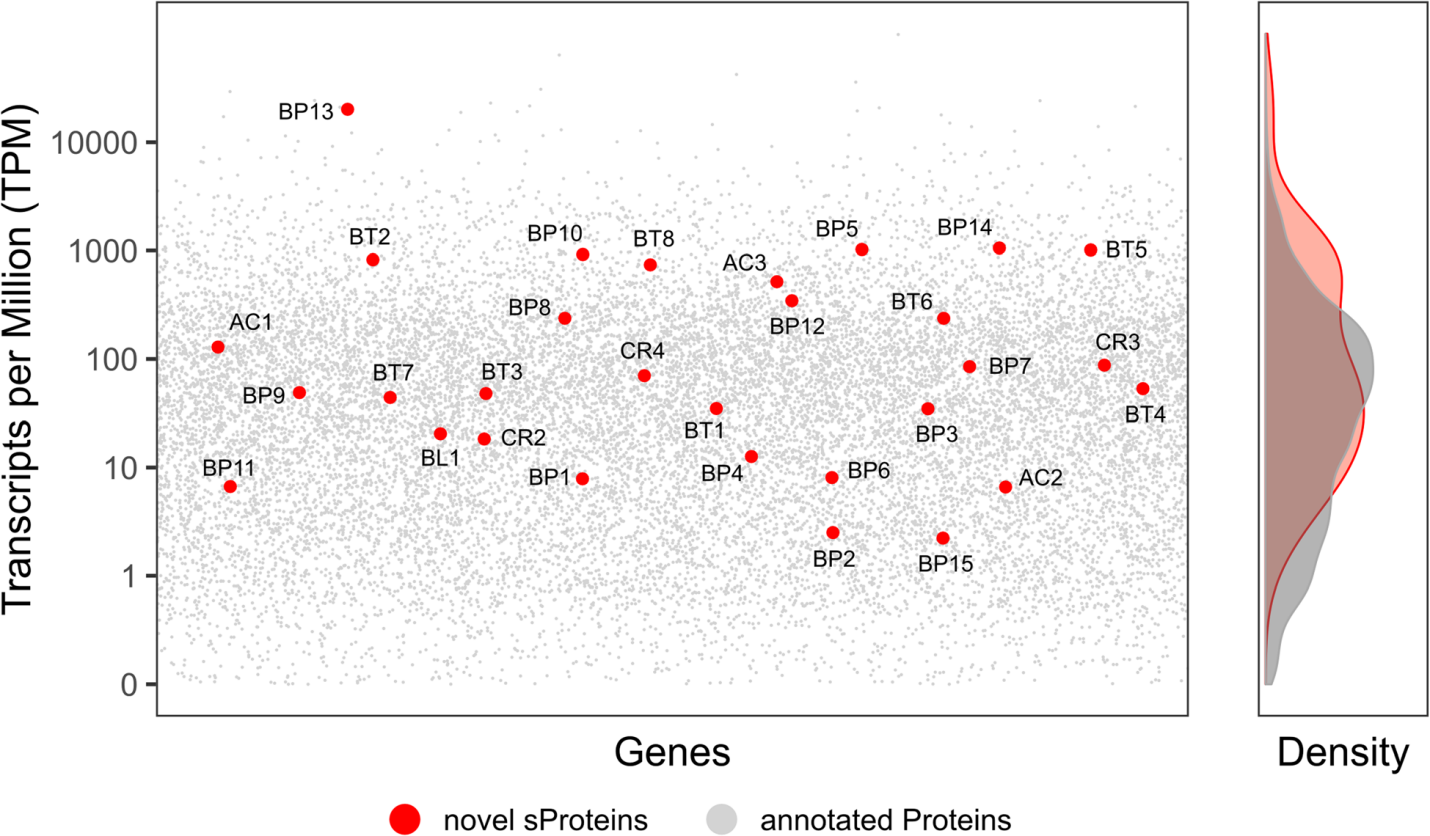
Shows the average of transcripts per million (TPM) normalized gene counts (in log-scale) and the density distribution of annotated and novel sProteins after metatranscriptome sequencing of the SIHUMIx community. After calculation of TPMs for each sample and species separately, all four samples have been averaged for visibility reasons. TPMs for the sProteins located in duplicated regions (BP4 and BP14) were averaged

Metatranscriptome sequencing of the SIHUMIx community was performed to assess whether the expression data for the genes encoding the respective novel sProteins supported their identification at the protein level. Thereby, transcriptomic evidence for 30 out of 31 novel sProteins was detected. The corresponding gene models for the two novel sProteins (BP4, BP14) exclusively identified by 3a peptides, are localized in duplicated regions; hence, the average of multi-mapping reads was added to the number of uniquely mapped reads. The abundance of novel sProtein gene transcripts were ranging from lower levels (6 sProteins below a TPM of 10) to high levels (6 sProteins with a TPM around 1000) indicated by the red dots in Fig. 5 while the pseudogene BP13 shows the highest expression level. Several factors may prevent the identification of proteins even with highly transcribed genes [60], but overall, there is a good correlation between gene expression levels and protein identification rate [60, 61].

### Validation of novel sProteins

**Fig. 6:**
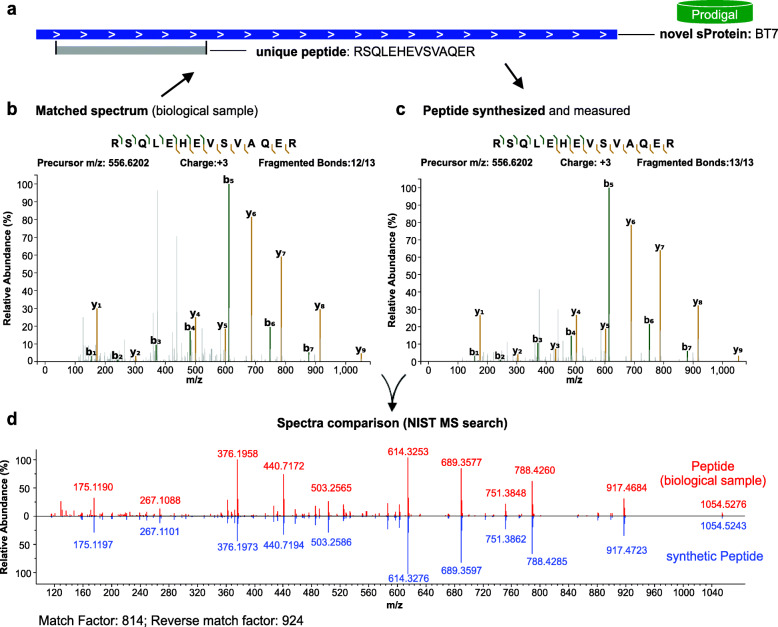
Validation of sProtein identification with synthetic peptides. **a** The novel sProtein BT6 (57 aa) predicted by Prodigal and detected with the unique, fully tryptic peptide RSQLEHEVSVAQER is shown, as well as **b** the MS/MS spectrum which was assigned to the peptide spectrum match (PSM) by the search algorithm. **c** The unique peptide was synthesized and a MS/MS spectrum was generated. **d** Both spectra were compared using NIST’s MS search software, which resulted in a match factor of 814 and reverse match factor of 924, well above the cut-off values, thereby confirming expression of the novel sProtein BT6

To validate the expression of the novel sProteins, the corresponding PSMs were further examined. For each identified peptide uniquely assigned to a sProtein, a synthetic peptide was tested. Mass spectra were acquired for the synthetic peptides and then compared with the MS/MS spectra assigned to the PSMs using NIST MS search, as described before [62]. If both spectra showed a high level of identity with a match score ≥ 500 and reverse match score ≥ 700, the PSM was considered verified as shown for the novel sProtein BT6 encoded by *B. thetaiotaomicron* (Fig. 6). If one unique peptide per sProtein was verified, the sProtein was considered validated. With this strategy, 25 out of 31 novel sProteins were validated (Supplement Figure 3). Among 6 candidates that could not be validated by spectra comparison, only one was rejected based on the score thresholds. The remaining 5 could not be validated because the synthetic peptides could not be ionized and therefore no spectra could be acquired for spectra comparison.

### Novel sProteins: SIHUMIx community vs. single strain cultivation

To analyze a potential community associated function, we investigated the protein expression of our 25 validated novel sProteins in SIHUMIx, which were cultivated as single strains. The single strains were harvested in growth phase and processed using the same proteomic protocols that led to the identification of the novel sProteins. However, only trypsin was used as a proteolytic enzyme. The MS/MS data was again searched against the species-specific iPtgxDBs using SequestHT and MS-GF+. Using the same stringent multi-step filtering criteria as described above, we were able to identify and confirm the expression of 18 novel sProteins that had been identified in the community culture and which were also expressed in the single-strain cultures. One novel sProtein (CR1) could not be compared, because it was identified using Asp-N as proteolytic enzyme. Interestingly, 6 novel sProteins (BP3, BP5, BP8, BP11, BP12, CR2) were uniquely expressed in the SIHUMIx community (Table 4).

**Table 4:** Physicochemical, functional, and structural prediction of novel sProteins exclusively identified in the SIHUMIx community culture experiments.

Abbr.	Size [aa]	p***I***	Aliphatic index	Gravy score	AMPs (prediction probability)	Localization (posterior probability)	Structure prediction
CR2	31	10.95	62.9	− 1.2968	AMP (0.992)	Cytoplasmic (0.646)	
BP3	45	6.11	56.4	− 1.7111	Non-AMP (0.002)	Cytoplasmic (0.534)	
BP5	48	9.87	48.8	− 1.7083	Non-AMP (0.001)	Non-cytoplasmic (0.539)	
BP8	57	10.01	80.4	− 0.5320	Non-AMP (0.002)	Non-cytoplasmic (0.663)	
BP11	64	6.68	68.4	− 0.6781	Non-AMP (0.053)	Non-cytoplasmic (0.596)	
BP12	66	9.64	70.9	− 0.6409	AMP (0.986)	Non-cytoplasmic (0.634)	

### Characteristics of identified SIHUMIx sProteins

Characterization of the six novel sProteins exclusively identified in the SIHUMIx community (Table 4) revealed that the majority of them (BP5, BP8, BP12, CR2) exhibited a high p*I* (> 8.0), likely resulting in a strong positive net-charge of these molecules. The p*I* of the other two sProteins (BP3 and BP11) was close to 7.0. Together with the negative GRAVY score of all sProteins, indicating a hydrophilic character, this points towards good water solubility of these candidate sProteins. Despite the fact that no signal peptides were predicted for any of these protein sequences, the AMP Scanner software [49] predicted antimicrobial peptide activity for CR2 and BP12, which usually occur in free solution. Many of these proteins are unstructured upon interaction with biological membranes [63], which fits well to the structure prediction of CR2. The physicochemical parameters and functional predictions of all novel sProteins are listed in Supplement Table 6.

### Potential role of sProteins in microbial community metabolism

We used metabolic microbial community modelling to elucidate a potential association between sProteins differentially abundant in community vs. single culture growth and enzymes that can be found adjacent to them in the genome. To this end, we used gapseq to reconstruct metabolic networks from the sequenced genomes of each strain and used constraint-based metabolic modelling to study differences in metabolic activity between single culture and community growth (Supplement Table 7). Thereby, we predicted metabolic activities (i.e., reaction fluxes) for each species either growing in isolation or in community.

**Fig. 7:**
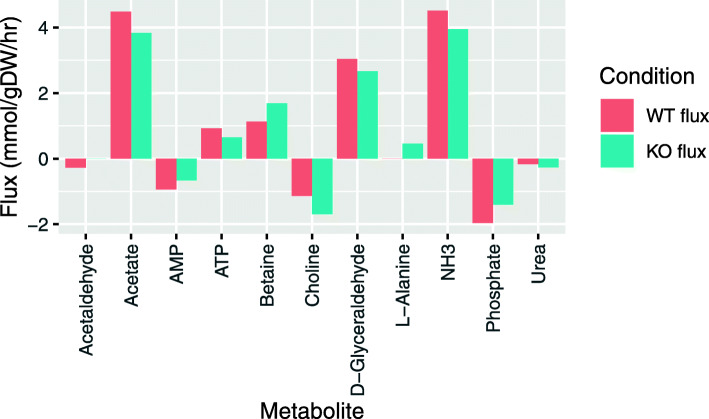
Role of enzymes in the close genomic vicinity of BP5 in the interaction of *B. producta* with SIHUMIx. Changes in the predicted metabolite exchange by *B. producta* with other member species of the community following knockout of rxn15467. Negative values indicate uptake of a compound by *B. producta* and positive values a production

Among the six sProteins that showed differential abundance in community vs. single culture growth, four were located within a 15,000-bp window of seven enzymes that were active in single culture or community growth (see Supplement Table 8). These enzymes participate in ten reactions, five of which show differential activity between single culture and community growth. Of these five, all occurred close to the sProtein BP5 in the metabolic network of *B. producta*. For three of them, it was predicted that they are active in single culture growth (“rxn00003,” “rxn00203,” and “rxn00898”) but inactive in community growth while two are inactive in single culture growth but active in community growth (“rxn15021” and “rxn15467”). These enzymes are part of the isoleucine biosynthesis from threonine and the TCA cycle. To further investigate the role of the enzymes activated in community growth, we re-performed the community modelling after an in silico knockout of the corresponding reactions (see “Material and methods”). In particular after knockout of rxn15467, a (R)-2,3-dihydroxy-3-methylbutanoate hydrolyase that catalyzes the second-to-last step in valine biosynthesis, we observed a considerable change in the predicted patterns of metabolite exchanges between *B. producta* and the other bacterial strains in the microbial community (Fig. 7). Knockout of rxn15467 was predicted to lead to an overall reduction of the production of metabolites for other community members by *B. producta*, while the consumption of metabolites was increased. Thus, production of the short-chain fatty acid acetate as well as production of ATP from AMP was reduced. Moreover, the uptake of choline along with the production of betaine was increased. These simulation results support the notion that BP5 and the enzymes located in its genomic neighborhood play an important role in the interaction between *B. producta* and the other member species of SIHUMIx.

## Discussion

sProteins carry out numerous important functions [14, 16, 64]. Historically, they have often been overlooked, as appropriate experimental enrichment strategies are required for their identification [19, 65, 66] and due to a number of computational challenges. These challenges include the low number of unambigous and MS/MS detectable peptides per sProtein, the need to apply stringent FDR cut-offs (see below) and, even more fundamentally, the unsolved problem of accurate and comprehensive ab initio gene prediction. While the minimal length cutoffs (between 50 and 100 aa for CDSs) applied by most gene prediction tools effectively minimize the inclusion of spurious short ORFs [15], they do miss a number of truly coding sProtein genes. Advances in proteomics and ribosome profiling [67], the two major technologies for a large-scale identification of missing sProteins, have further fueled the interest in this important protein class both in bacteria [21, 68, 69] and in humans [70]. Notably, Sberro et al. predicted thousands of novel sProteins based on a metagenomics study of human-associated microbiomes, several of which play important roles in host-microbiome and bacteria-bacteria interactions [18]. Using metaproteomics, they could detect 25 sProteins [18]. The challenges in detecting sProteins using standard proteomics protocols, the lack of an adjusted protein search database and the intra- and inter-species redundancy of protein sequences may have hindered the identification of a representative number of sProteins in complex microbial communities [19, 71].

The combination of proteogenomics with metaproteomics (metaproteogenomics) has recently been shown to be a valuable tool for microbiome research [72]. To target novel community-relevant sProteins, we here applied a metaproteogenomic approach on a defined moderately complex model community of the human gut, consisting of eight bacterial species. For this, we extended our previously developed proteogenomics approach to identify novel sProteins in a single prokaryote [21] to the SIHUMIx model system. We first created complete genomes for 6 SIHUMIx strains for which only fragmented genome assemblies existed. This approach provides an optimal basis for comprehensive sProtein discovery and downstream functional genomics. Recently, it had even identified several essential genes missed in an incomplete assembly of Pseudomonas aeruginosa MPAO1 [73], the widely used parental strain of a transposon insertion library. The careful hierarchical integration of reference genome annotations, ab initio gene predictions and in silico predictions into a minimally redundant yet highly informative iPtgxDB ensured that 90% or more of all MS/MS identifiable peptides uniquely point to one protein entry in the search database (a class 1a peptide) [40]. This percentage amounted to 93% in the combined, eight species iPtgxDB (Supplement Table 9) and largely facilitates downstream data analysis. The optimized, lean database structure that captures the entire protein coding potential of a completely sequenced genome is crucial to reduce type II error in peptide identifications, because of reduced FDR sensitivity for large protein databases, as usually encountered in metaproteomics [74–76].

We applied both a stringent, multi-tiered FDR control [21], suggested by [20], as well as subsequent validation steps (see below). The PSM FDR level was set to result in a 1% protein FDR. This set-up resulted in 6576 total protein identifications and 904 sProteins of which 253 sProteins were not contained in the NCBI RefSeq annotation. We additionally required 2, 3, or 4 PSM hits for a reference annotation, Prodigal and ChemGenome ab initio gene prediction, or an in silico gene prediction, respectively, i.e., increasing evidence for less reliable prediction sources. Accordingly, this step reduced the number of novel sProteins from 253 to 31, effectively removing “one-hit wonders” (proteins identified with 1 peptide and a single spectrum). Notably, Prodigal contributed most novel sProteins, but also ChemGenome and the in silico predictions added some novel sProtein identifications (e.g., CR2, see below), re-confirming the value of our integrative approach. A less stringent filtering could in principle also be used, as long as a downstream validation of all peptides implying a novel sProtein is carried out, which also becomes a cost issue.

*B. thetaiotaomicron*, one member of our SIHUMIx community, was investigated by Sberro and colleagues using single culture proteomics. Out of 35 sProteins < 50 aa predicted with high confidence, they were able to identify 4 using proteomics. Our SIHUMIx metaproteomics data confirmed 2 of those, while one additional novel sProtein was only identified with 1 PSM in our study and was thus filtered out. We identified an additional eight novel sProteins in *B. thetaiotaomicron*, which was likely due to our enrichment for sProteins, being able to search against a comprehensive iPtgxDB (based on complete genomes) and using a different size threshold for sProteins [18]. The fact that several of these missed sProteins were > 50 aa supports the selection of a threshold of 100 aa for a comprehensive discovery of novel sProteins.

Our 25 validated novel sProteins showed a wide range of physicochemical properties (Supplement Table 6) which suggests a great diversity of potential functions. Eighteen of those novel sProteins are predicted to be non-cytoplasmatic or transmembrane. Since cell-cell and cell-host communication is often mediated by small diffusible molecules secreted by cells or by direct cell-cell contact, these proteins may be involved in cell-cell communication [77, 78]. A functional protein domain prediction (Prosite) indicated that the 3 novel sProteins AC2, BT5, and BT8 contain a potential Big-1 domain (bacterial Ig-like domain 1) (Supplement Table 6). Big-1 proteins are surface-expressed proteins that mediate mammalian host cell invasion or attachment in enteropathogenic bacteria [79–81]. This domain is part of adhesion molecules of the intimin/invasin family. Furthermore, it has been shown that Big-domain-containing protein InvD from *Yersinia pseudotuberculosis* acts by binding the Fab region of IgG or IgA and might therefore avoid the clearance from the intestine by secretory IgA, making these proteins interesting targets to study bacteria-host interactions [82]. Although the SIHUMIx species are not enteropathogenic, these three novel sProteins are interesting candidates for the study of host microbiome interactions. Additionally, we found domains involved in carbohydrate metabolism as for example the mannose 6-phosphate receptor homology (MRH) domain in BP11 [83], or the FtsK domain involved in cell division in AC1 and CR4 [84]. Another interesting novel sProtein is BP10 containing a potential MarR-type HTH domain, which is involved in the development of antibiotic resistance [85, 86].

Recent studies indicate that sProteins play an important role in multi-species communities [18, 87]. In the context of this work, we have identified 18 novel sProteins in the SIHUMIx community and single cultures, which can be interpreted as further layer of validation. Nevertheless, six novel sProteins could only be identified in the community, which potentially suggests a possible community-associated function. However, it should be noted that growth and cultivation conditions differ between individual cultures and communities and are difficult to control. The novel proteins can therefore also be attributed to these varying conditions. Most of these sProteins are predicted to be non-cytoplasmatic, indicating a role outside of the cell or membrane association. This further promotes the chances of being directly involved in cell-cell communication. Interestingly, an antimicrobial function was predicted for two (CR2 and BP12) of the six novel sProteins. AMPs are small, have cationic, amphiphilic, or hydrophobic properties, which make them interact with the negatively charged bacterial membrane on which they form pores that cause cell death. In bacteria, the production of AMPs represents a competitive advantage thus ensuring their survival in the community in ecological niches [88]. We tested the two AMP-predicted sProteins, CR2 and BP12 (ranked among novel sProteins with the 3rd and 5th highest AMP prediction score; data not shown), on the growth of SIHUMIx species (Supplement Figure 6). Interestingly, only for *C. butyricum* we observed a significant extension of the lag time while grown on 1 μM synthetic CR2 sProtein. This result may imply a specific interspecies interaction between the two *Clostridium* strains. It has been previously reported that *Clostridia* interact with each other [89] and also on the basis of AMPs [90].

These AMPs can further alter the normal bacterial flora of the gastrointestinal tract to allow colonization and proliferation of *Clostridia* [91]. It further supports that most bacterial AMPs have a very narrow target spectrum, i.e., they are only active against a few species closely related to the producer [92, 93]. These finding may explain the relatively low abundance of *C. butyricum* in the SIHUMIx community during our in vitro bioreactor cultivations (Fig. 4). Notably, we observed a significant reduction in cell size after treatment of *C. butyricum* with CR2 (Supplement Figure 7). Such a property has already been described for other AMPs and thus supports the prediction of an antimicrobial effect of CR2 [94, 95]*.* However, more experiments are needed to verify and further analyze this function. Also, other novel sProteins, which had an even higher predicted AMP prediction score, are interesting candidates for investigation.

Using metabolic modelling, we also investigated the importance of enzymes found in the genomic environment of our novel sProteins. In particular, we analyzed those enzymes that play a role in metabolic interaction within the SIHUMIx community. We observed that BP5, a 48-aa-long novel sProtein only identified in community cultivation, is located in a genomic region that contains several enzymes whose activity is relevant for community metabolism (Supplement Table 8). Those enzymes were part of the isoleucine biosynthesis from threonine and the TCA cycle. An in silico knockout of (R)-2,3-dihydroxy-3-methylbutanoate hydrolyase which is in close genomic neighborhood to BP5 and catalyzes the second-to-last step in valine biosynthesis, led to a reduced metabolite production and concurrently increased metabolite consumption by *B. producta* for the SIHUMIx community members. This indicates an important function for community metabolism and leads to the hypothesis that BP5 may serve as potential mediator or modulator of community interactions in SIHUMIx. Future experiments, e.g., knock-out studies of BP5, are needed to verify this feature.

In summary, this study shows that proteogenomics can be used with metaproteomics to improve genome annotation and to provide a better interpretation of microbiome data. As these sProteins play a potentially important role in prokaryotic microbial communities, we recommend that future bacteria and microbiome studies systematically analyze sProteins (including potential novel sProteins) which is facilitated by the public available iPtgxDB web server.

## Conclusions

Our study shows that an integrated proteogenomic approach for the discovery of novel sProteins is applicable to microbial communities. In total, we identified 31 novel sProteins, of which we were able to validate 25. The comparison to protein expression in single strains showed that 6 novel sProteins could only be identified in the bacterial community, indicating a potentially important community-related function of these sProteins. Further in silico studies and experiments showed that one of these novel sProteins had a potential antimicrobial function and one sProtein likely being involved in community-related metabolism making these candidates particularly interesting for further studies on intestinal community shaping.

## Supplementary Information

**Additional file 1. Supplement.**

**Additional file 2. Supplement Table 4.**

**Additional file 3. Supplement Table 6.**

**Additional file 4. Supplement Table 8.**

**Additional file 5. Supplement Table 9.**

## Data Availability

PacBio, ONT, and Illumina data were uploaded to NCBI’s short read archive (SRA) and can be accessed via the following BioProject and sequence accession numbers: PRJNA523317, CP036345 (*A. caccae*); PRJNA531376, CP039126 (*B. producta*); PRJNA523323, CP036346 (*C. ramosum*); PRJNA531377, CP039121 and CP039122 (*L. plantarum*); PRJNA543750, CP040530 and CP040529 (*B. thetaiotaomicron*); PRJNA544389, CP040626, to CP040629 (*C. butyricum*). The iPtgxDBs can be downloaded from https://iptgxdb.expasy.org/. Metatranscriptomics data can be accessed via the following Bioproject: PRJNA655119; proteomics data (both from individual single strain cultures and the SIHUMIx grown in the bioreactor) have been uploaded to PRIDE and can be assessed under PXD020005.

## Abbreviations

aa: Amino acid
AMP: Antimicrobial peptide
BHI: Brain heart infusion
bp: Base pairs
CIM: Complex intestinal media
COG: Cluster of orthologous groups
FASP: Filter aided sample preparation
FDR: False discovery rate
gDNA: Genomic DNA
GRAVY: Grand average of hydropathy
iPtgxDB: Integrated proteogenomics search database
LC: Liquid chromatography
MS/MS: Tandem mass spectrometry
NSAF: Normalized spectral abundance factor
ONT: Oxford Nanopore Technology
ORF: Open reading frame
p*I*: Isoelectric point
PSM: Peptide spectrum match
SIHUMIx: Simplified human intestinal microbiota
SP3: Solid-phase-enhanced sample preparation
sProtein: Small protein
TPM: Transcript per million
