# Supplementary material for: Discovery of novel community-relevant small proteins in a simplified human intestinal microbiome
Source: Microbiome. 2021 Feb 23;9:55. doi: 10.1186/s40168-020-00981-z (PMC7903761; doi:10.1186/s40168-020-00981-z)
Supplement: Supplementary file 2 — Additional file 1. Supplement. [file 40168_2020_981_MOESM2_ESM.docx]

Additional file 1: Supplement Figure 1
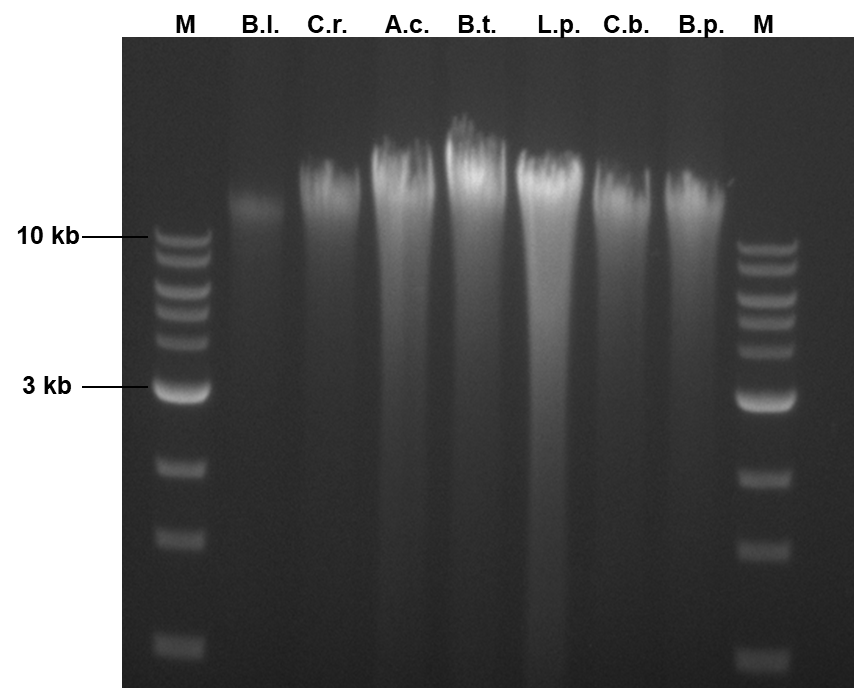


**Supplement Figure 1**: Agarose gel electrophoresis of isolated genomic DNA of *B. longum* (B.l.), *C. ramosum* (C.r.), *A. caccae* (A.c.), *B. thetaiotaomicron* (B.t.), *L. plantarum* (L.p.), *C. butyricum* (C.b.) and *B. producta* (B.p.).

Additional file 1: Supplement Figure 2

| 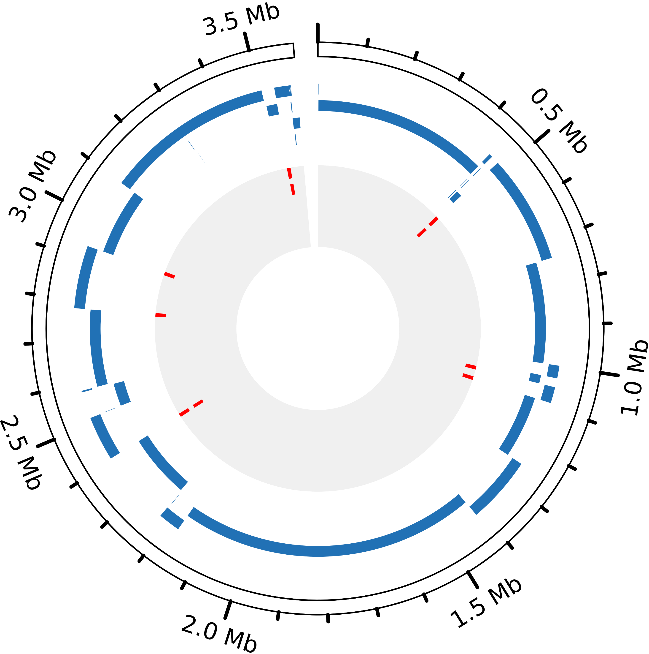  **a** | 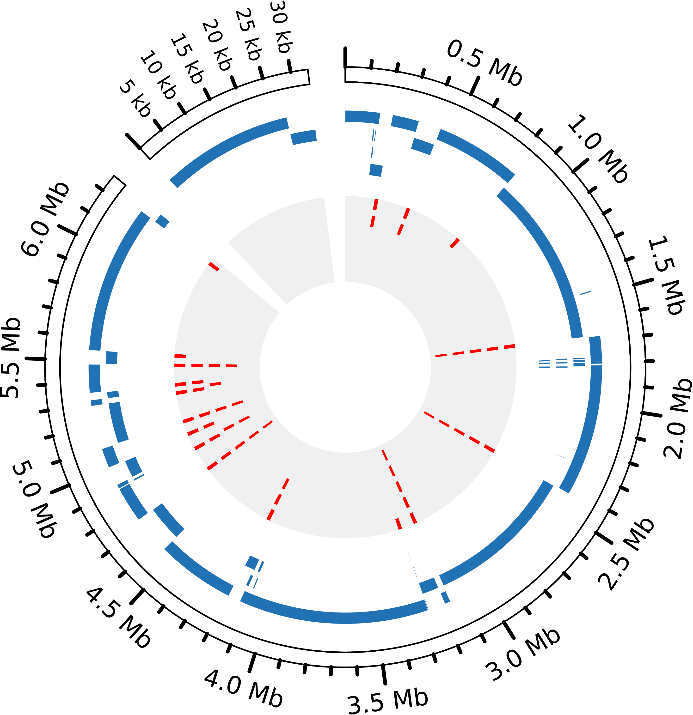  **b** |
| --- | --- |
|  |  |
| 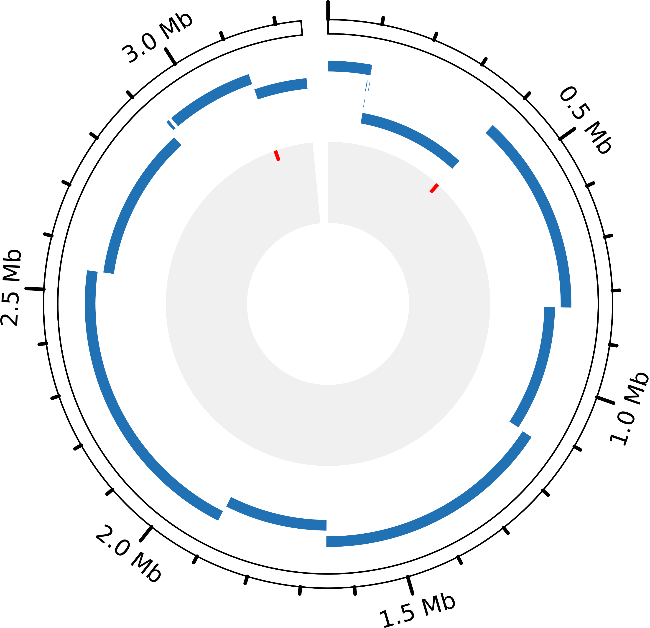  **d**  **c** | 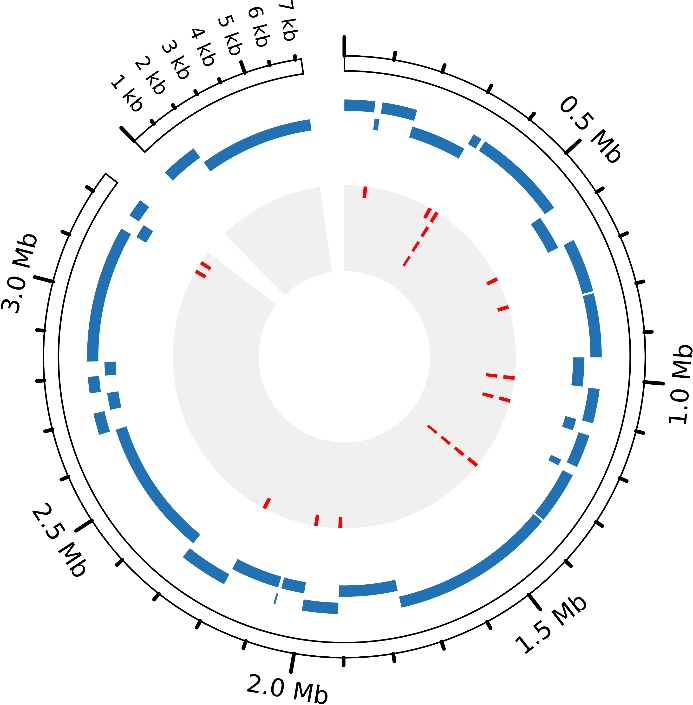 |

**Supplement Figure 2:** Comparison of complete, long read-based *de novo* assembled genomes (outer circle showing the size of the bacterial chromosome and plasmids) and the corresponding fragmented, short read-based assemblies available at RefSeq (blue contigs). The positions of missed genes are marked in red on inner grey shading. **a)** *A. caccae*; **b)** *B. thetaiotaomicron*; **c)** *C. ramosum*; **d)** *L. plantarum*.

Additional file 1: Supplement Figure 3

AC1

Peptide: MINYREELK


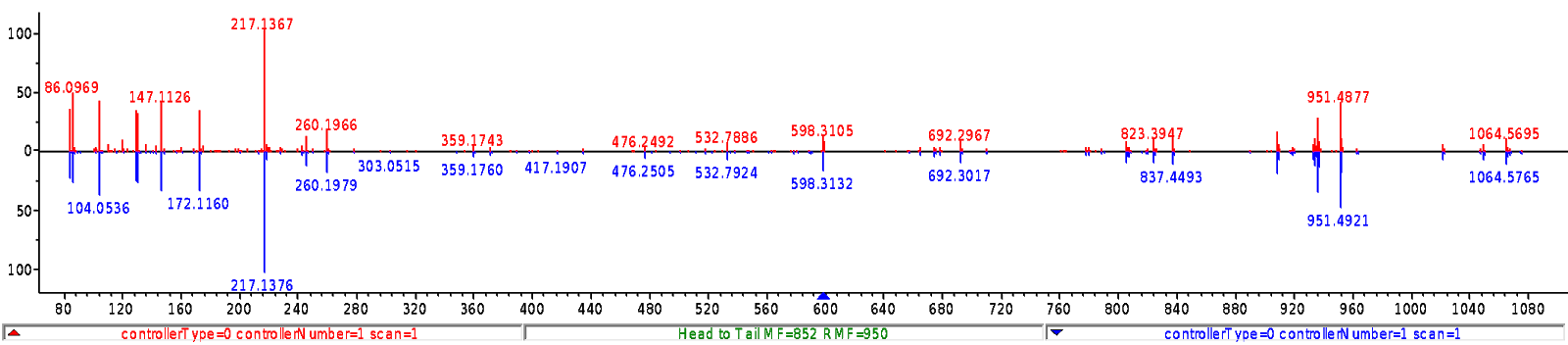


Match Score: 852; Reverse match score: 950

AC2

Peptide: FFLDTANVDEIR


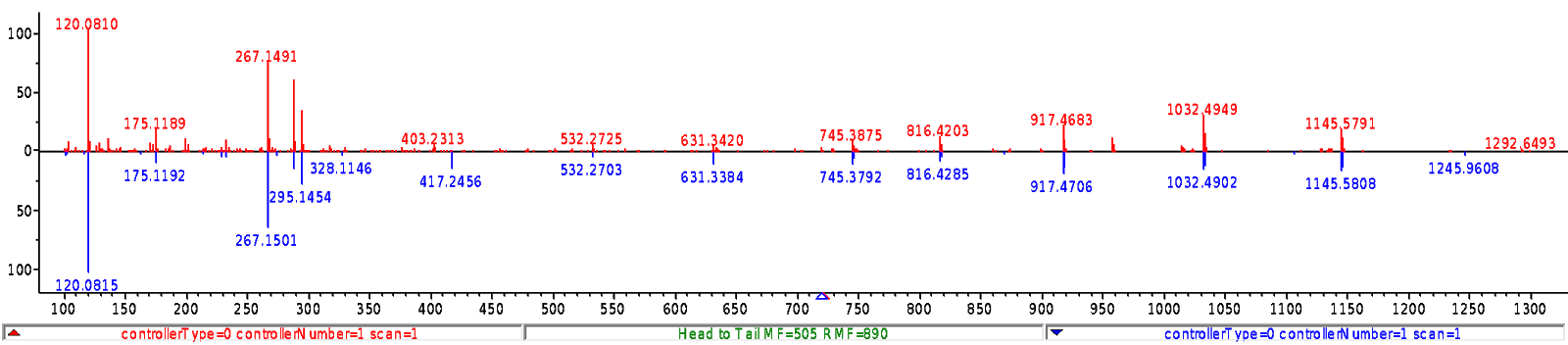


Match Score: 505; Reverse match score: 890

AC3

Peptide: AAFSYAGLEEATEK


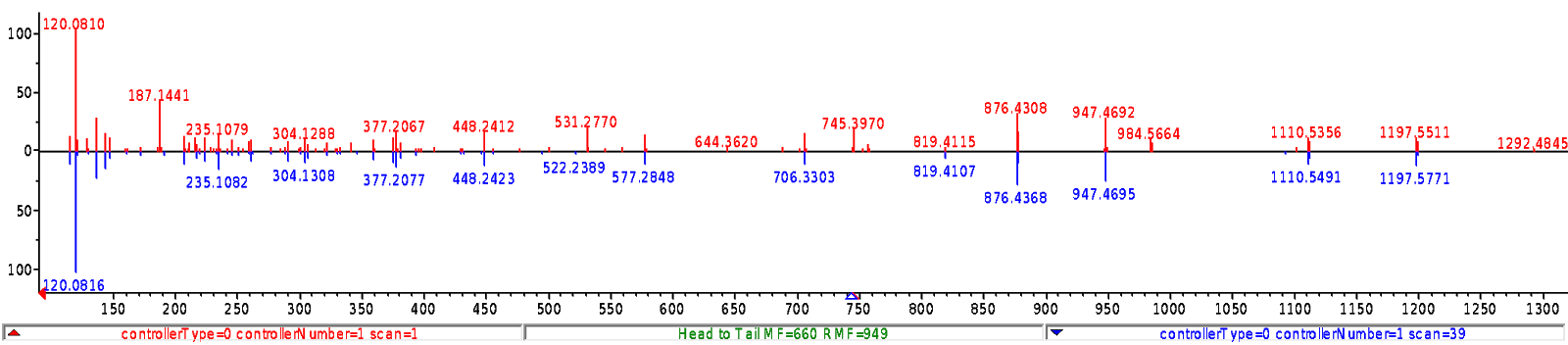


Match Score: 660; Reverse match score: 949

BP1

Peptide: LLVNAPEDK


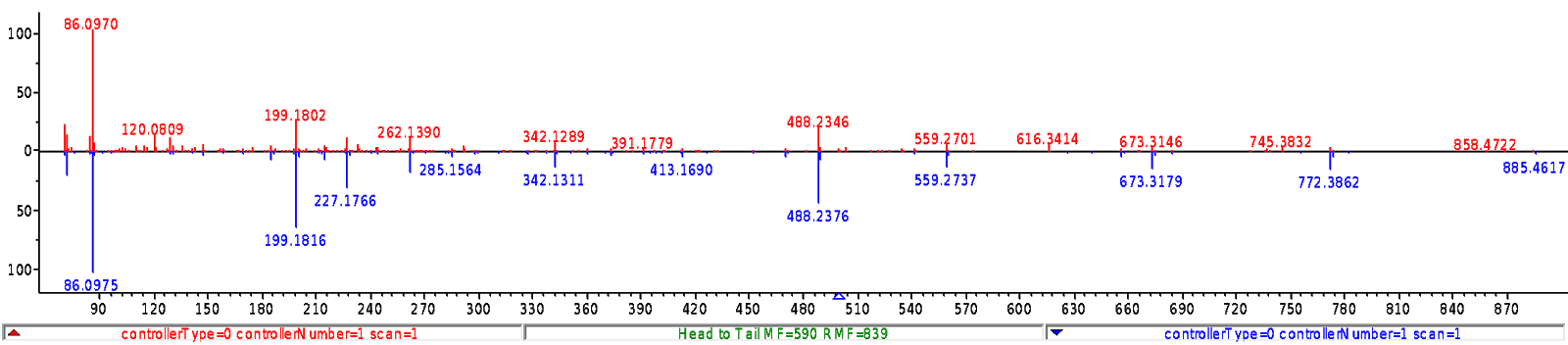


Match Score: 590; Reverse match score: 839

BP3

Peptide: VWREEPLR


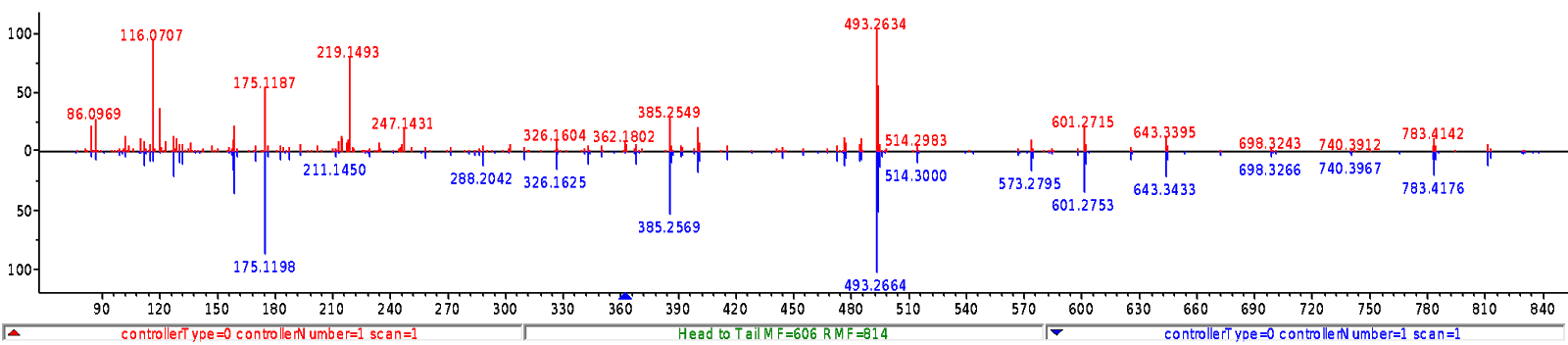


Match Score: 606; Reverse match score: 814

BP4

Peptide: DKQFKTILEMFGMIL


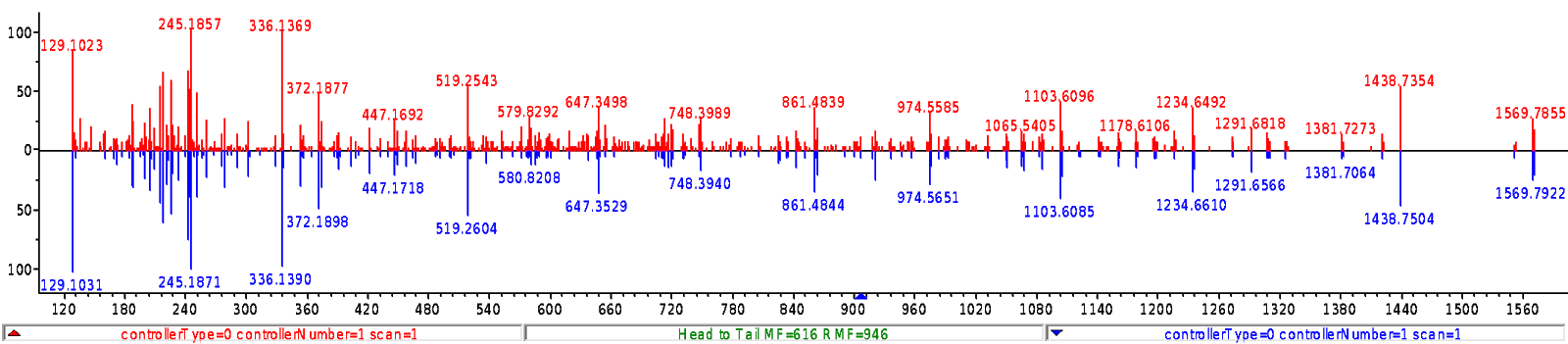


Match Score: 616; Reverse match score: 946

BP5

Peptide: SNETFNSITQK


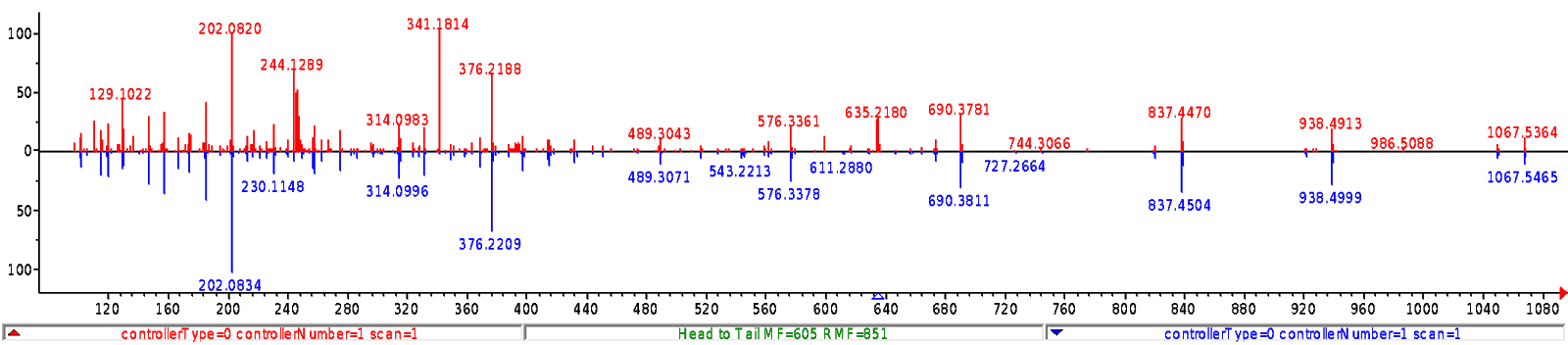


Match Score: 605; Reverse match score: 851

BP6

Peptide: QDLTDVIDILK


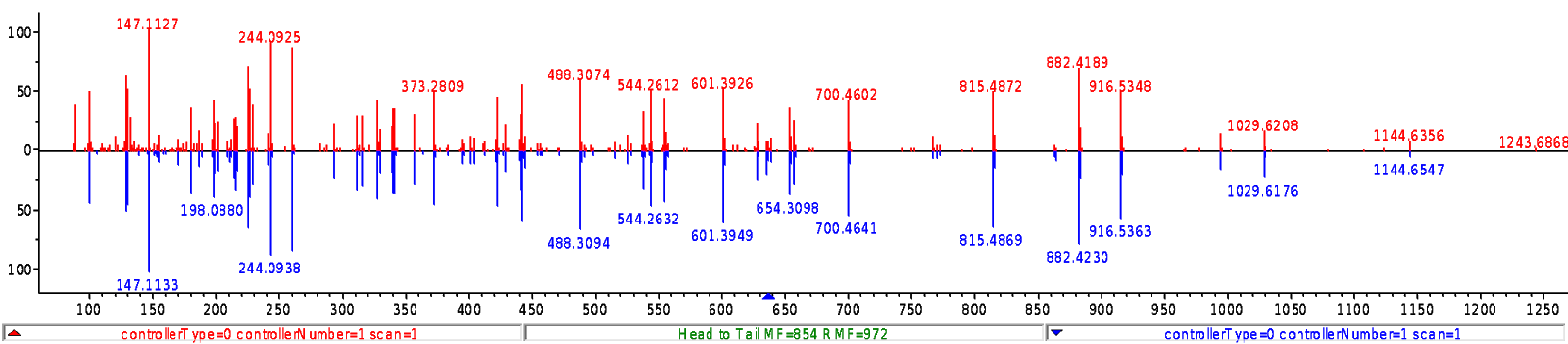


Match Score: 854; Reverse match score: 972

BP7

Peptide: LIDDTQKGIEDD


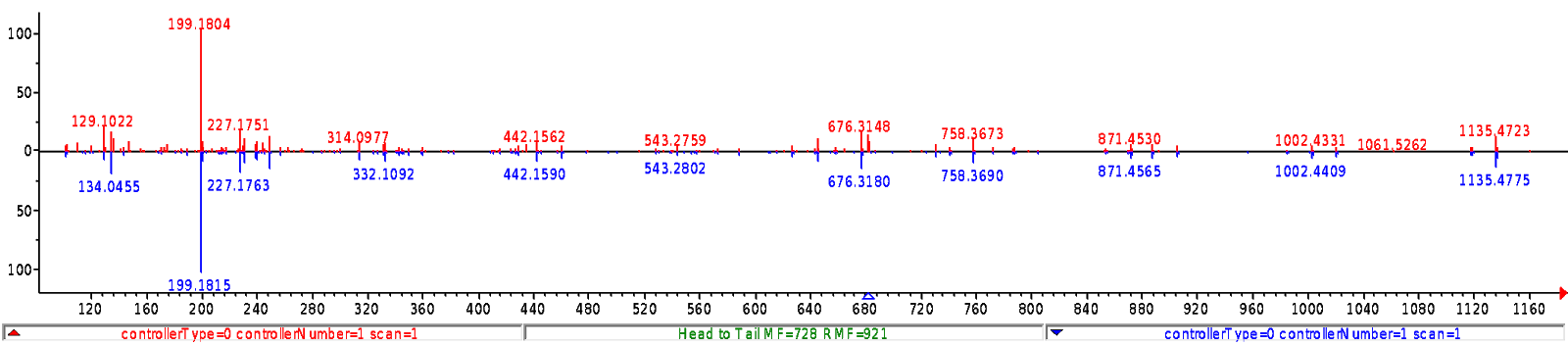


Match Score: 728; Reverse match score: 921

BP8

Peptide: KPLLAEAK


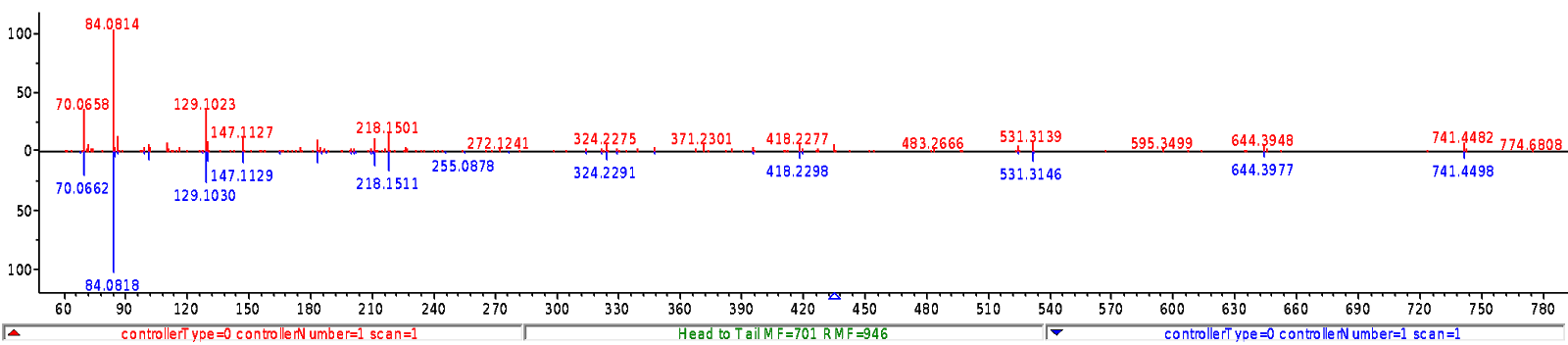


Match Score: 701; Reverse match score: 946

BP9

Peptide: SGETINGFIR


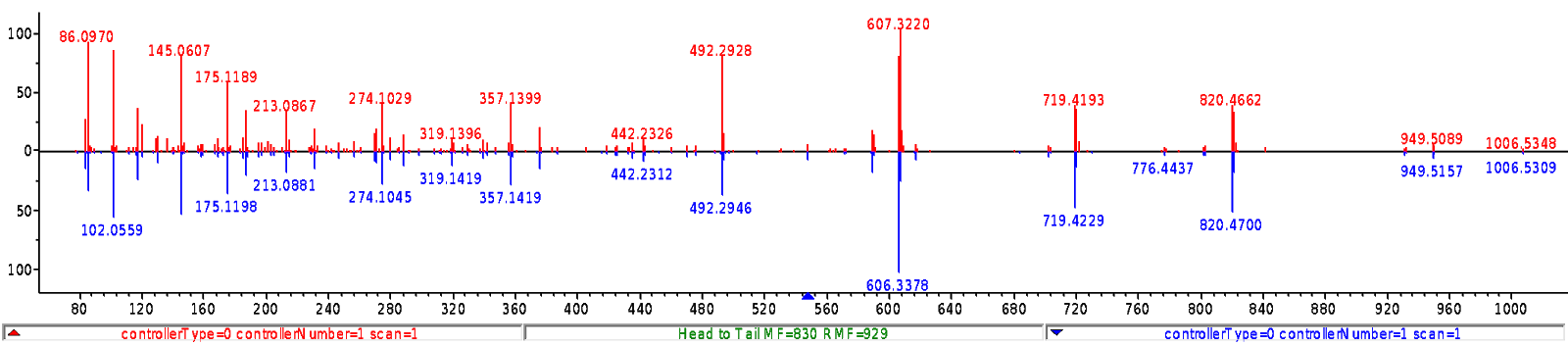


Match Score: 830; Reverse match score: 929

BP10

Peptide: DAYVQPTLRII


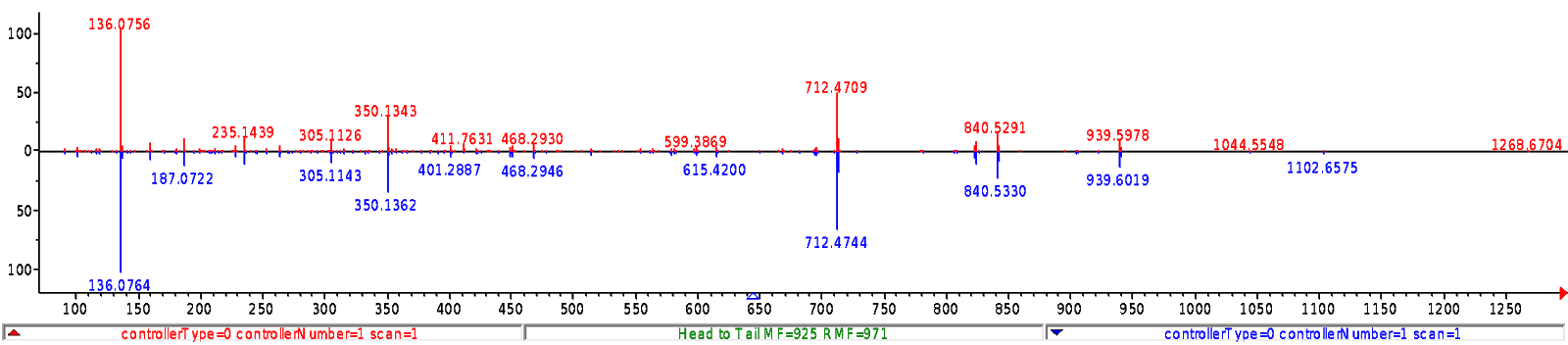


Match Score: 925; Reverse match score: 971

BP11

Peptide: NDAQTYTVVSQIK


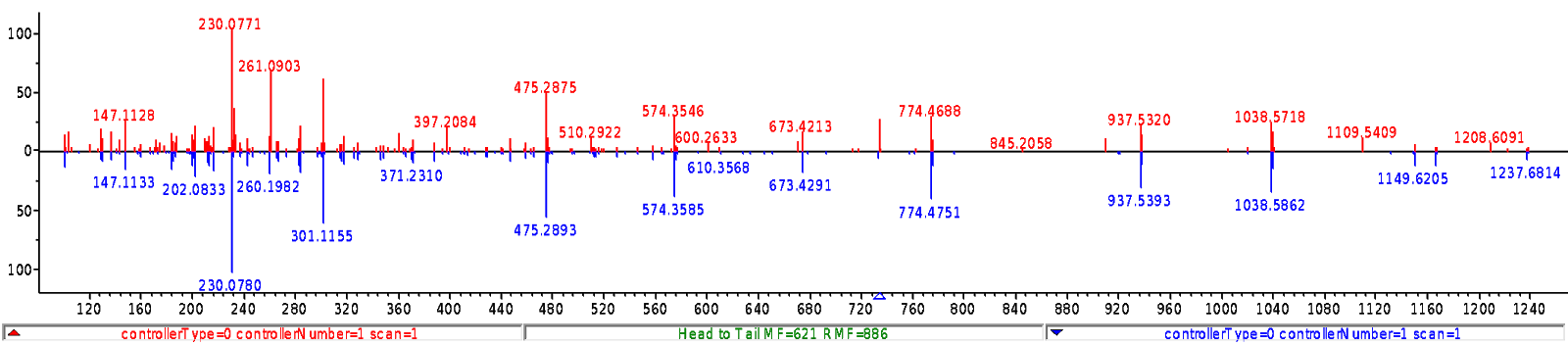


Match Score: 621; Reverse match score: 886

BP12

Peptide: DKRKLESAIK


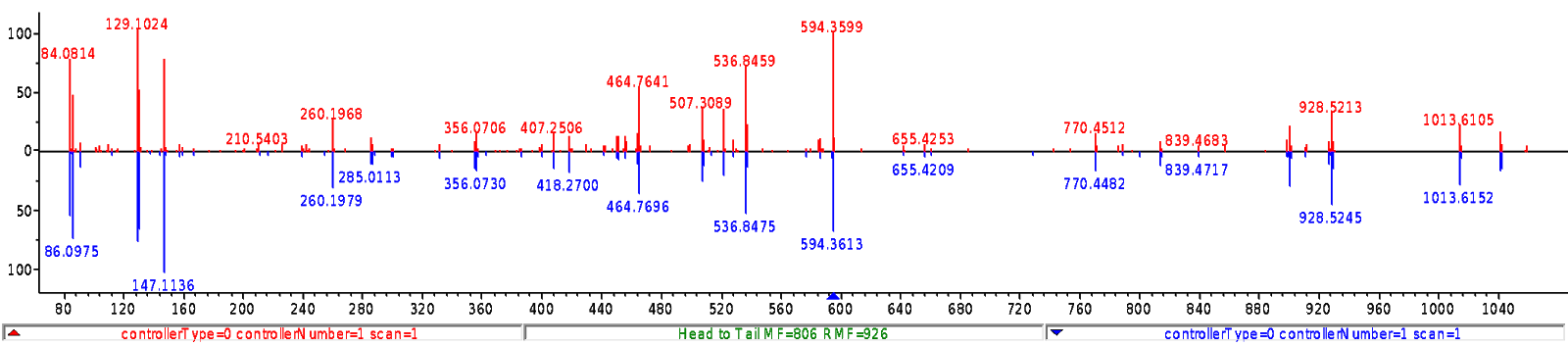


Match Score: 806; Reverse match score: 927

BP14

Peptide: TFASAIENASGR


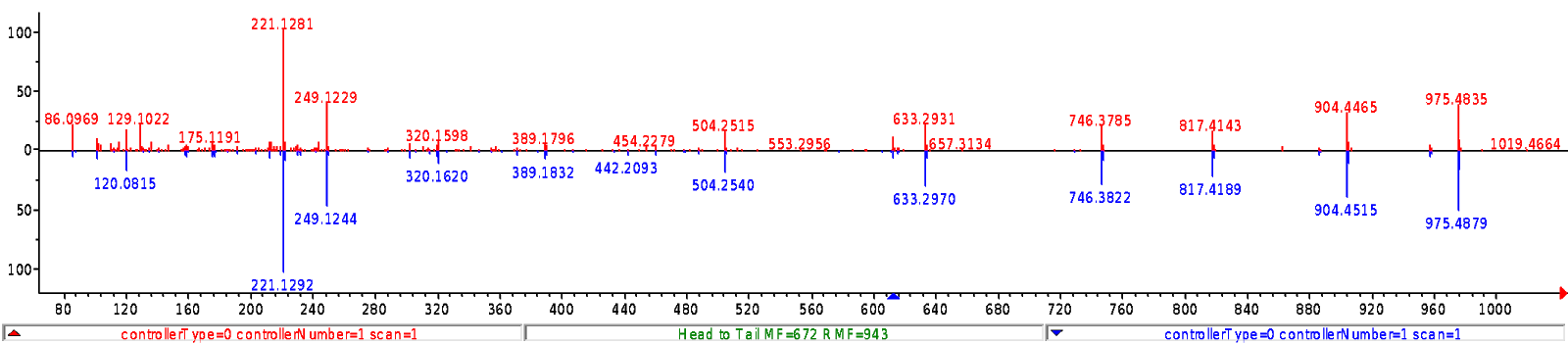


Match Score: 672; Reverse match score: 943

BT1

Peptide: IGNADVHVWGYSTLGILPK


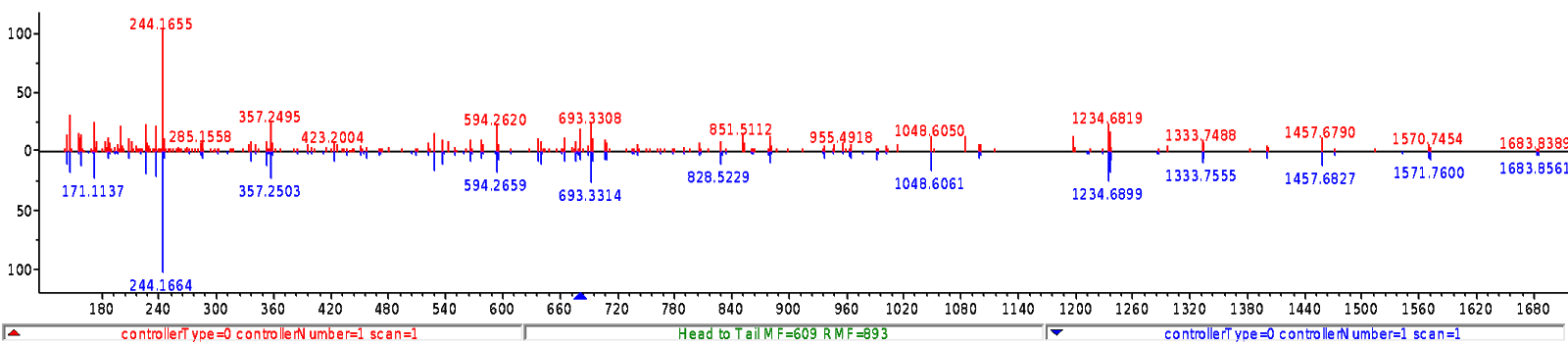


Match Score: 609; Reverse match score: 893

BT3

Peptide: MSSSMLEQR


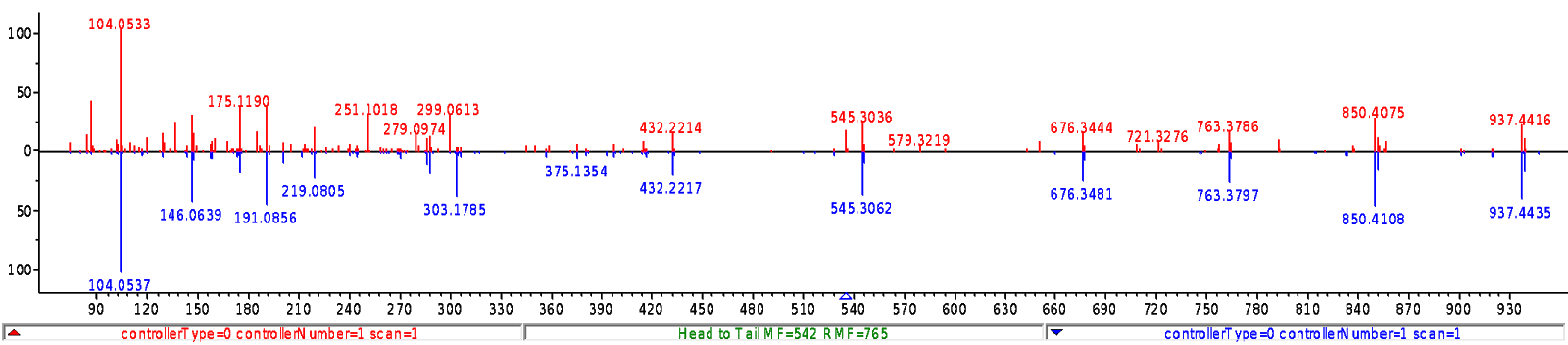


Match Score: 542; Reverse match score: 765

BT4

Peptide: NIPVLEGVTAEDFVR


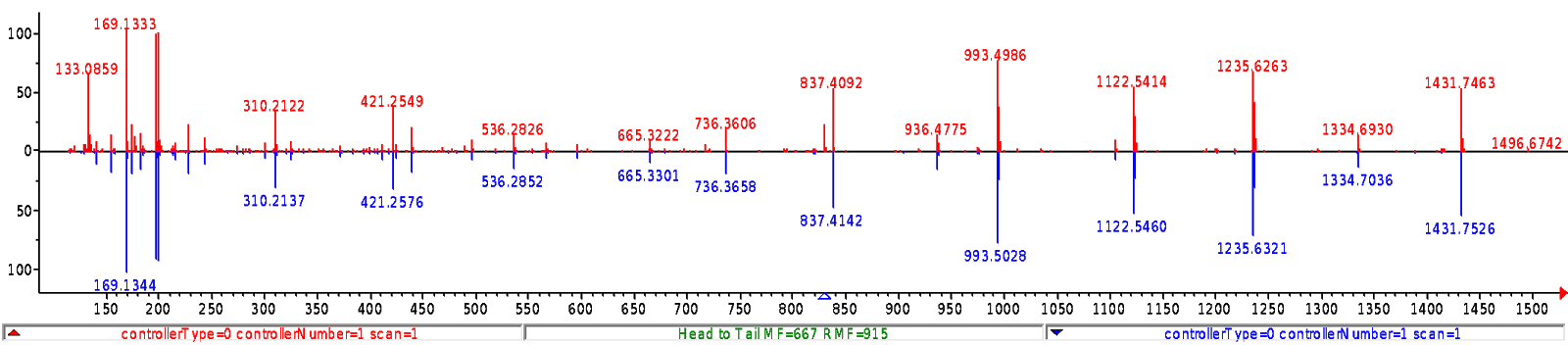


Match Score: 667; Reverse match score: 915

BT5

Peptide: MKNPPKETPEK


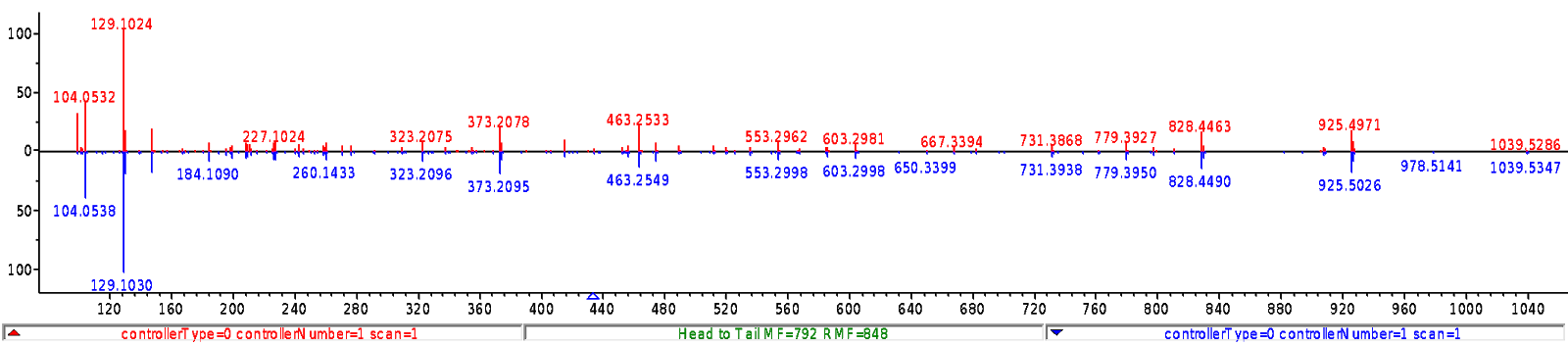


Match Score: 792; Reverse match score: 848

BT6

Peptide: DIIKLWEQ


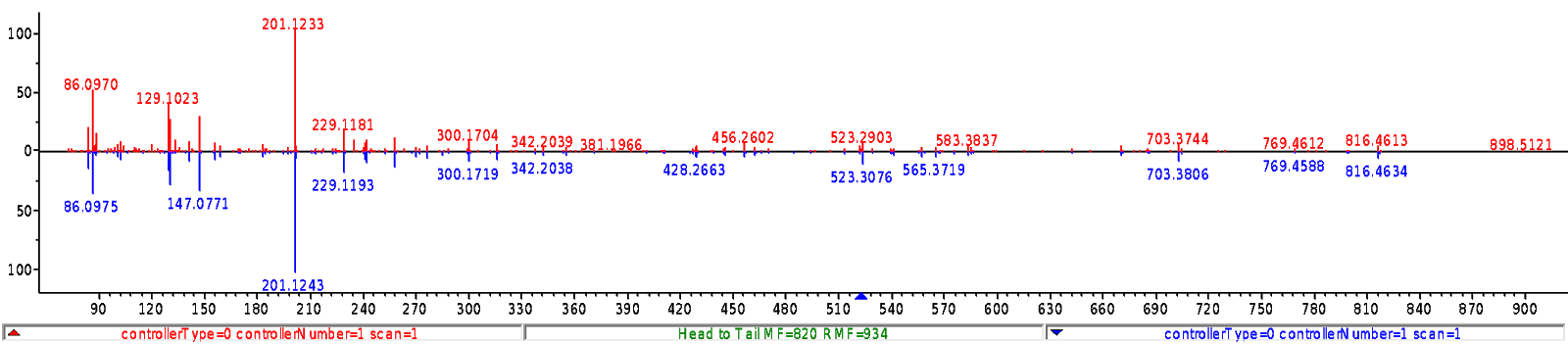


Match Score: 820; Reverse match score: 934

BT7

Peptide: DAIMGGIVFKGKK


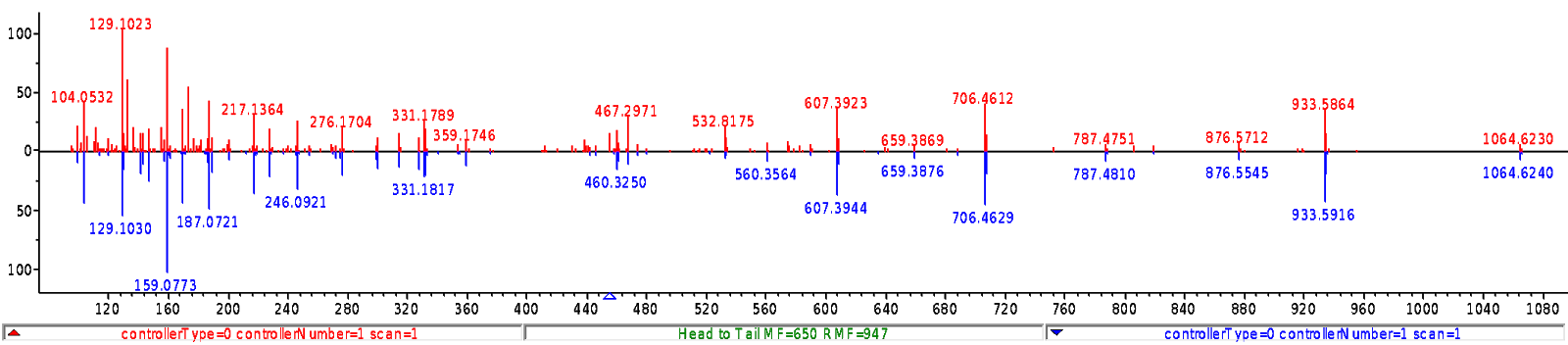


Match Score: 650; Reverse match score: 947

BT8

Peptide: MPQWLGTGVQKY


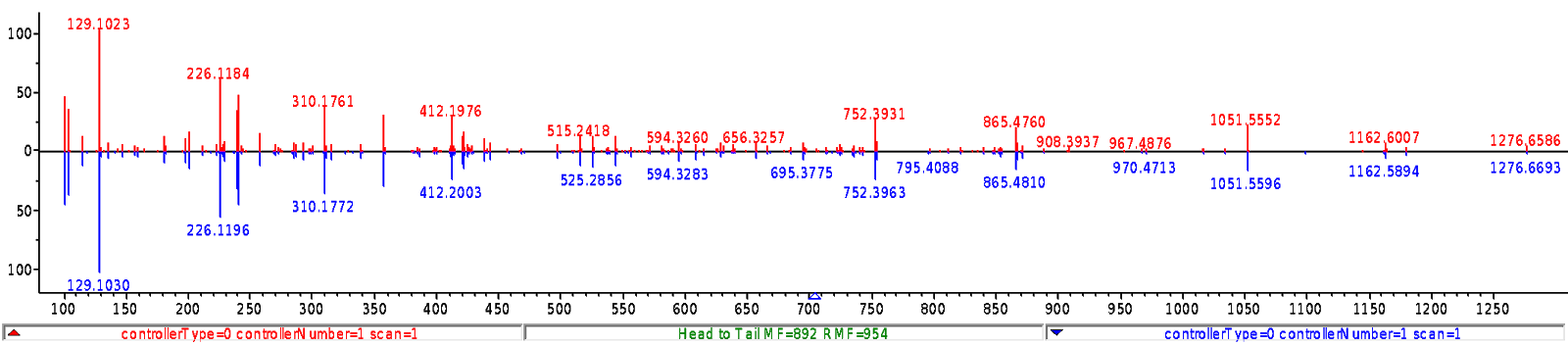


Match Score: 892; Reverse match score: 954

CR2

Peptide: IIKPIQPIKPSPPTK


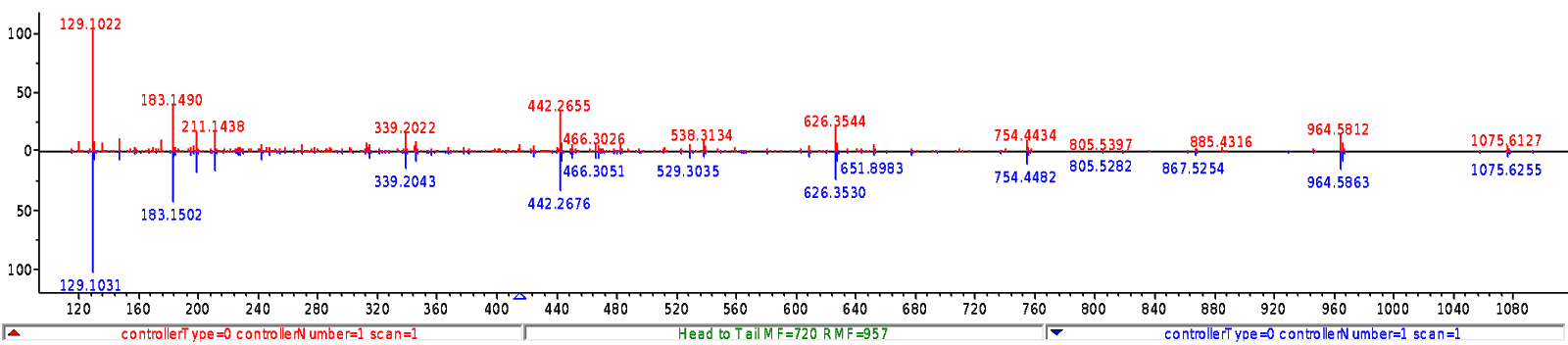


Match Score: 720; Reverse match score: 957

CR3

Peptide: DKIRALVKELF


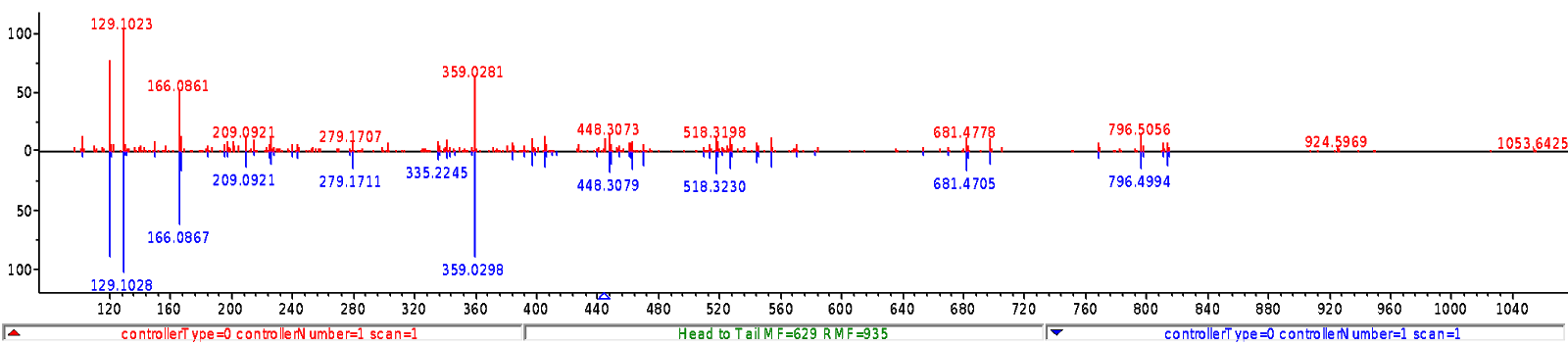


Match Score: 629; Reverse match score: 935

CR4

Peptide: LDEMIVLLK


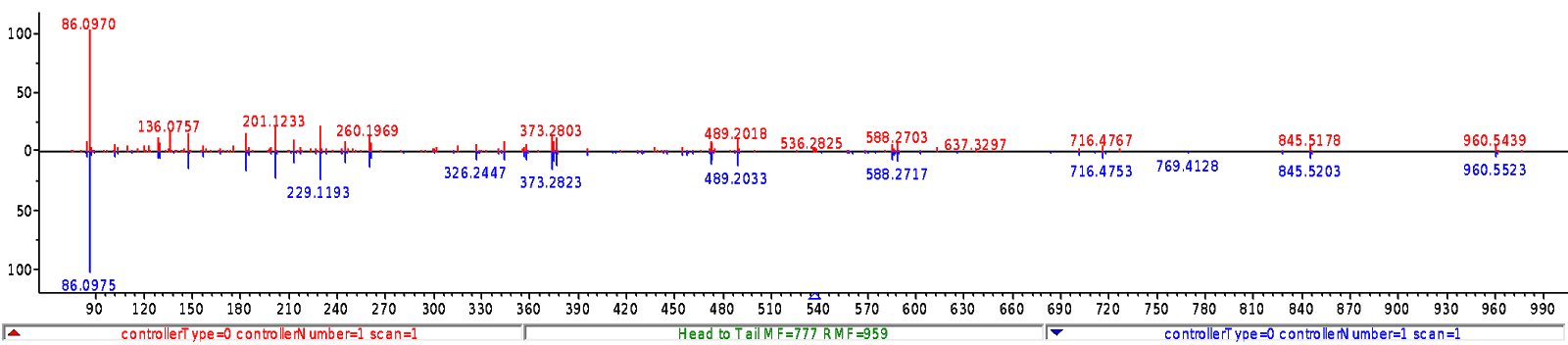


Match Score: 777; Reverse match score: 959

**Supplement Figure 3:** Comparison of spectra obtained for experimentally observed peptides and those acquired from synthetic peptides, analyzed with NIST MS Search Program (v.2.0g). Spectra were considered to be matching when they exhibited a match score of at least 500 and a reverse match score of at least 700.

Additional file 1: Supplement Figure 4


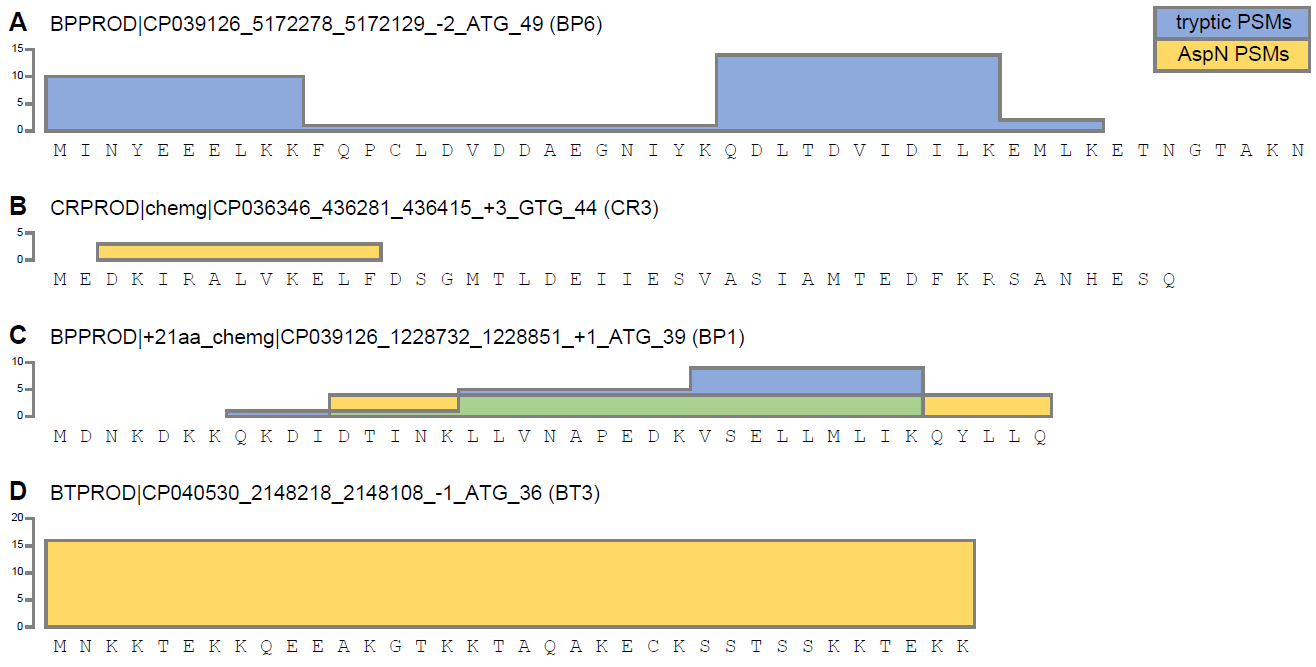


**Supplement Figure 4:** PSM distribution of selected novel sProteins; **a** BP6, exclusively detected by tryptic peptides; **b** CR3: exclusively detected by Asp-N peptides; **c** BP1, detected by tryptic and Asp-N peptides, with overlapping coverage; **d** BT3: directly detected by undigested protein (found in Asp-N sample preparation); These four examples further illustrate the coding of the identifier generated while hierarchical integration of different annotation resources. Examples **a**, **c** and **d** are predicted by Prodigal, an *ab initio* gene prediction software (Hyatt et al., 2010), while example **b** was in addition also predicted by Chemgenome (Singhal et al., 2008). The identifier encodes the prediction source, the chromosome/plasmid and coordinates, the frame, the start codon and the length of the protein. For more information how to read these identifiers, see: https://iptgxdb.expasy.org; color code: blue: tryptic PSMs, yellow: Asp-N PSMs, green: overlap of tryptic and Asp-N PSMs.

Additional file 1: Supplement Figure 5
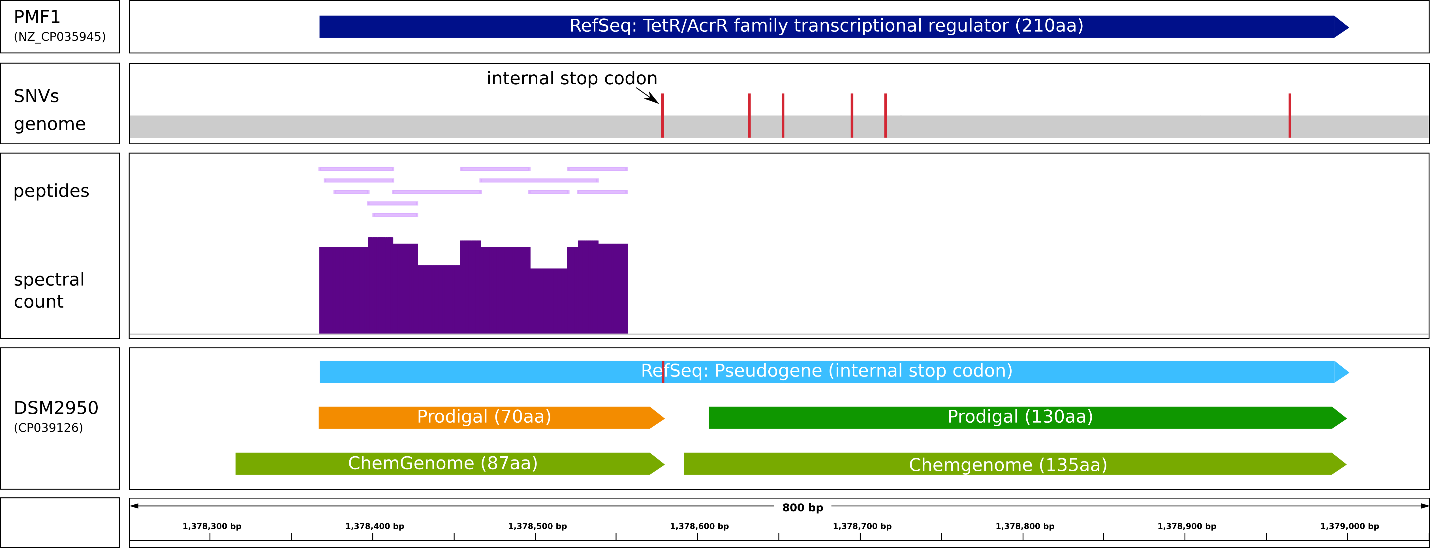


**Supplement Figure 5:** Comparsion of *B. producta* strain PMF1 (NZ_CP035945) to our strain DSM2950 (CP039126). The observed N-terminal concentration of PSMs on the 70aa-Prodigal prediction indicates at least N-terminal expression of the annotated pseudogene TetR/AcrR family transcriptional regulator also in strain DSM2950.

Additional file 1: Supplement Figure 6

**
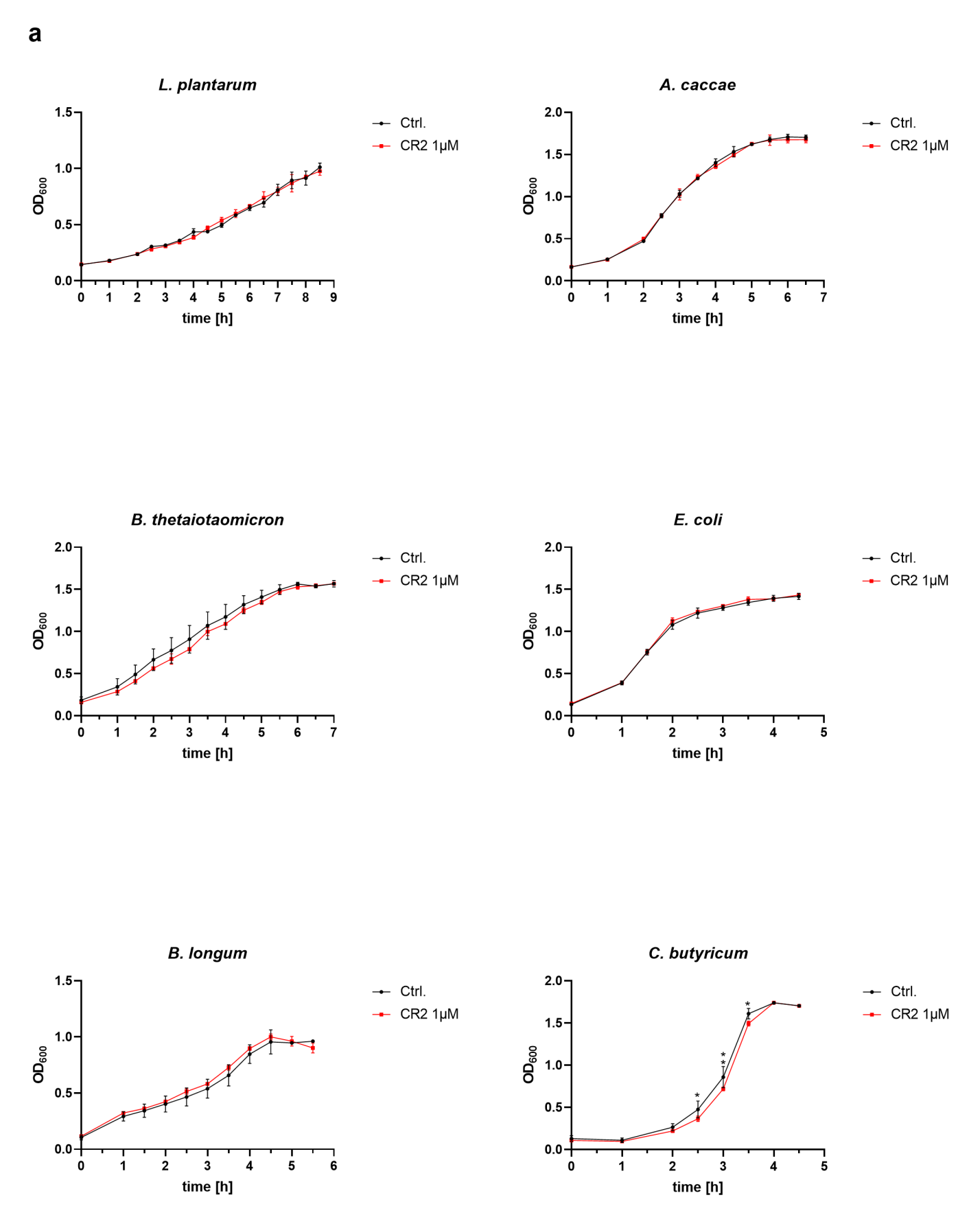
**

**
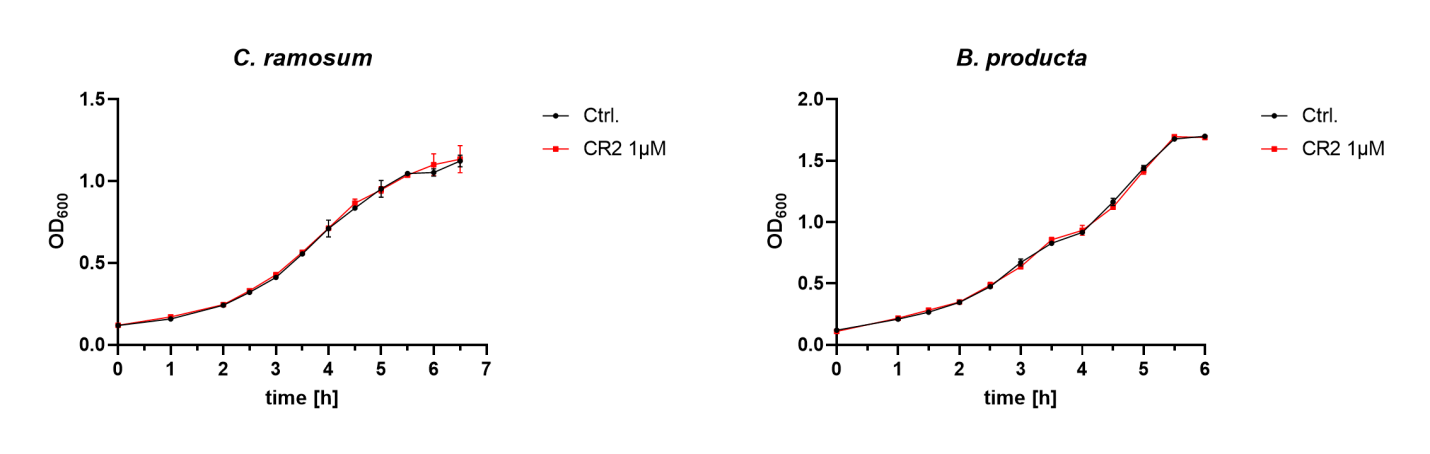
**

**
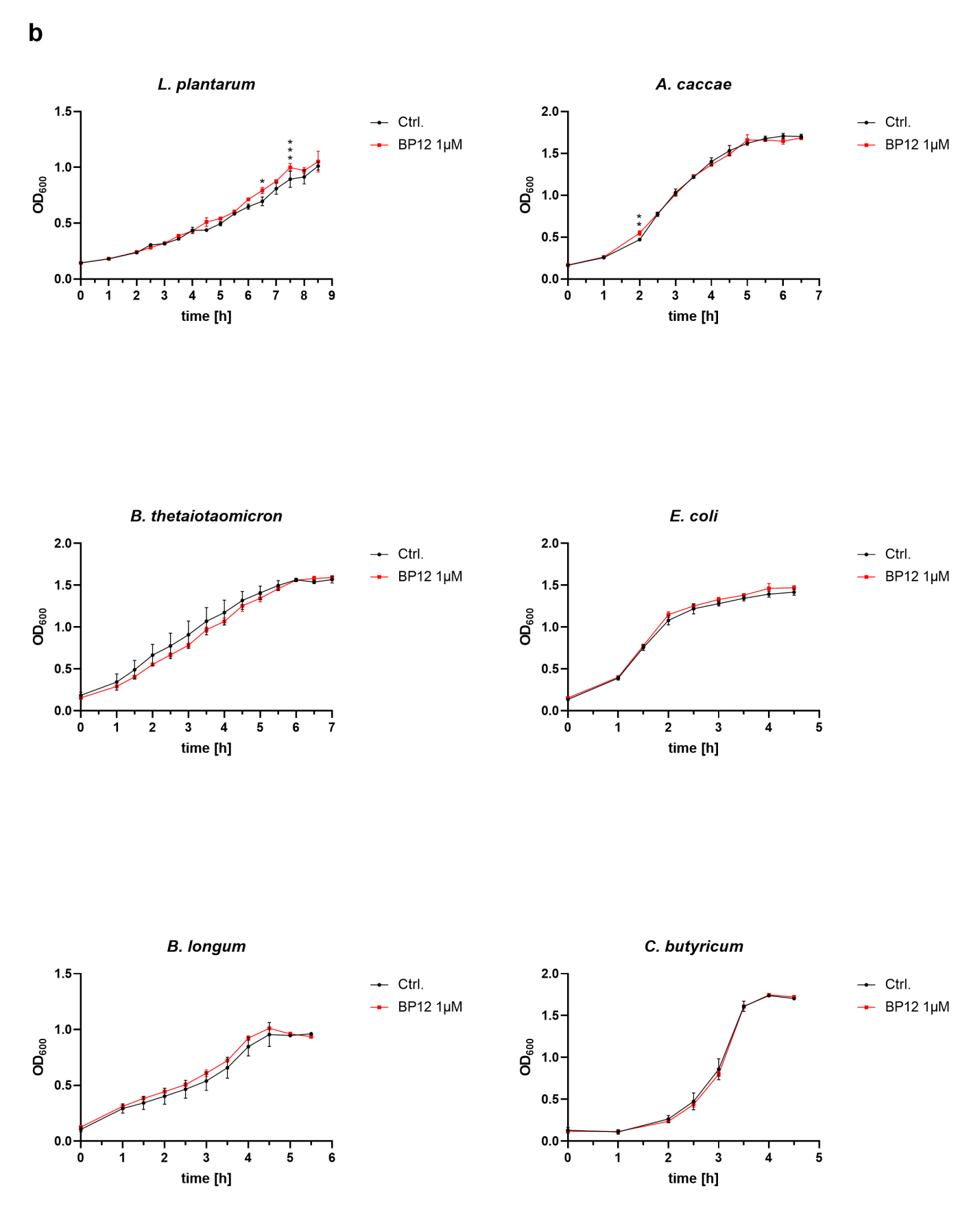
**

**
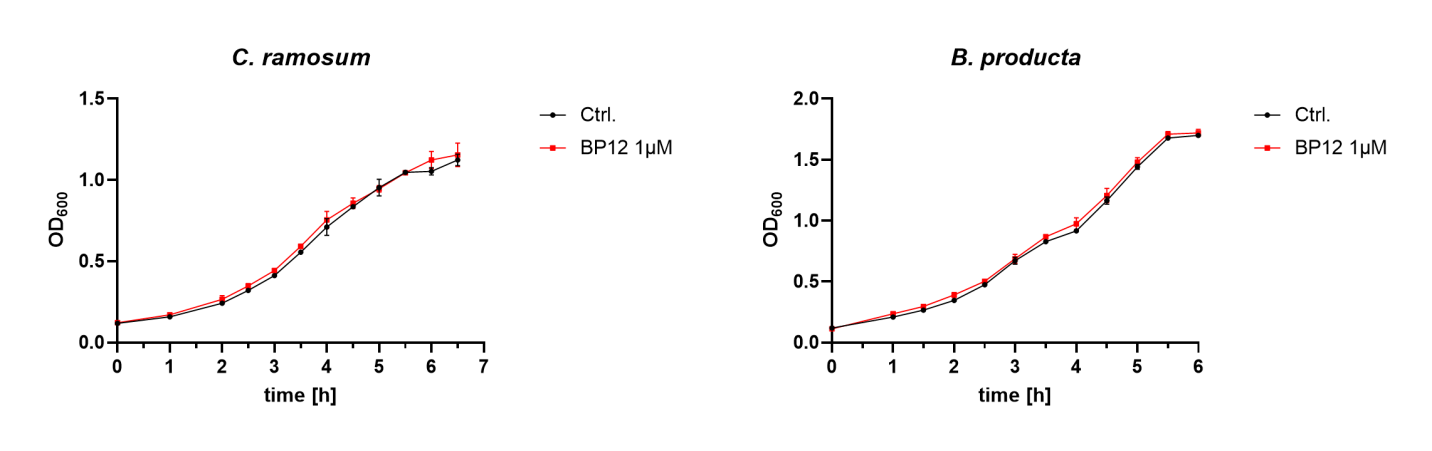
**

**Supplement Figure 6:** Growth curves of SIHUMIx species in single culture with treatment of potential novel detected AMPs. The SIHUMIx species *L. plantarum*, *A. caccae*, *B. thetaitaomicron*, *E. coli*, *B. longum*, *C. butyricum*, *C. ramosum* and *B. producta* were inoculated from an overnight culture in 4 mL BHI medium containing 100 µL sodium phosphate buffer (50 mM, 100 mM NaCl, pH 7) as control, or novel sProtein CR2 (a) or BP12 (b) dissolved in this buffer (final CR2 concentration 1 µM) and anearobically cultivated at 37°C and 175 rpm. Bacterial growth was measured by OD_600_ absorbance measurements at time intervals of 30-60 min. Statistical significance was determined in GraphPad Prism (V8.4.1) using the Holm-Sidak method, with alpha=0.05. Computations assumed that all rows are sampled from populations with the same scatter (SD); * p< 0.05; ** p< 0.01; *** p< 0.001; n=3.

Additional file 1: Supplement Figure 7


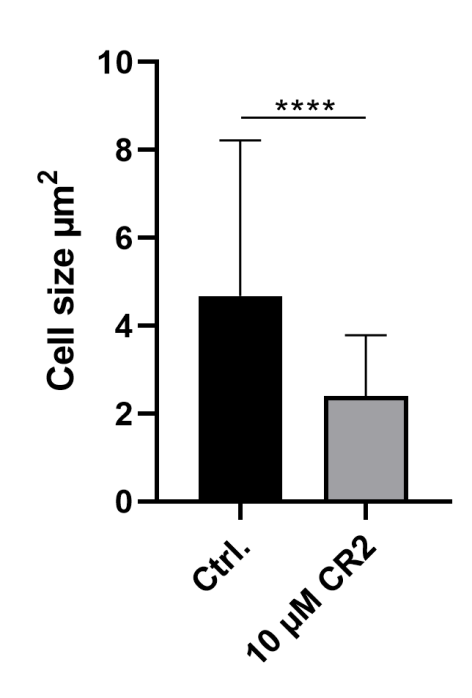


**Supplement Figure 7:** Cell size in µm^2^ of *C. butyricum* after 5 hour anaerobic cultivation in BHI medium (at 37°C and 175 rpm) containing 100 µL sodium phosphate buffer (50 mM, 100 mM NaCl, pH 7) as control, or novel sProtein CR2 dissolved in this buffer (final CR2 concentration 10 µM). Pictures of bacterial cells were taken with Quantom Tx^TM^ (Logos Biosystems, South Korea) after cell staining according to the manufacturer's instructions. Cell sizes were determined using ImageJ (v2)^1^ with Fiji plugin^2^. Control = 1,753 cells, 10 µM CR2 = 2,199 cells; statistical significance was determined with Mann-Whitney test (two tailed) in GraphPad Prism (V8.4.1); ****p<0.0001.

Additional file 1: Supplement Table 1

**Supplement Table 1:** Supplemented Brain-Heart-Infusion medium (BHI).

| Ingredient | Quantity [g or mL/L] | Supplier |
| --- | --- | --- |
| Brain-Heart-Infusion | 37 | Roth |
| L-cysteine hydrochloride | 0.5 | Biochemica |
| Resazurin | 0.001 | MP biomedicals |
| Vitamin K hemin solution | 10 | Becton Dickinson |
| Yeast extract | 5 | Chemsolute |

Additional file 1: Supplement Table 2

**Supplement Table 2:** Complex intestinal medium (CIM): Medium formulation was adopted from McDonald et al. (2013). PH was adjusted to pH 6.7 with NaOH. Storage at 4 °C

| **Ingredient** | **Quantity [g/L]** | **supplier** |
| --- | --- | --- |
| Arabinogalactan (larch wood) | 2 | Sigma-Aldrich |
| Bile Acids sodium salt | 0.5 | Sigma-Aldrich |
| Calcium chloride x 2 H_2_O | 0.01 | Merck |
| Casein peptone (pancreatic) | 4.3 | Roth |
| Di-Potassium hydrogen phosphate | 0.04 | Roth |
| Hemin (bovine) | 0.005 | Sigma-Aldrich |
| Inulin | 1 | Serva |
| L-cysteine hydrochloride | 0.5 | Biochemica |
| Magnesium sulfate | 0.01 | Roth |
| Menadione | 0.001 | Sigma-Aldrich |
| Mucin (porcine gastric Type II) | 4 | Sigma-Aldrich |
| Pectin, citrus peel | 2 | Sigma-Aldrich |
| Potassium di-hydrogen phosphate | 0.04 | Roth |
| Sodium chloride | 0.72 | Roth |
| Sodium hydrogen carbonate | 2 | Roth |
| Starch, wheat | 5 | Roth |
| Xylo-oligosaccharide (corn) | 2 | Roth |
| Yeast extract | 2 | Chemsolut |

Additional file 1: Supplement Table 3

**Supplement Table 3:** Genome assembly status of eight SIHUMIx strains. For two genomes, complete assemblies have been created, for six, only fragmented short-read based assemblies existed.

| **Phylum** | **Species** |  | **Strain** | **Gram** | **Contigs** | **RefSeq accession** |
| --- | --- | --- | --- | --- | --- | --- |
| Actinobacteria | Bifidobacterium *longum* | | NCC 2705 | + | 1* | NC_004307.2 / NC_004943.1 |
| Bacteroidetes | Bacteroides *thetaiotaomicron* | | DSM 2079 | - | 33 | GCA_900445595.1 |
| Firmicutes | Anaerostipes *caccae* | | DSM 14662 | +/- | 26 | GCA_000154305.1 |
| Firmicutes | Blautia *producta* | | DSM 2950 | + | 68 | GCA_000373885.1 |
| Firmicutes | Clostridium *butyricum* | | DSM 10702 | + | 207 | GCA_000409755.1 |
| Firmicutes | Lactobacillus *plantarum* | | DSM 20174 | + | 9 | GCA_000143745.1 |
| Firmicutes | Clostridium *ramosum* | | DSM 1402 | + | 12 | GCA_000154485.1 |
| Proteobacteria | Escherichia *coli* | | *K-12/*MG1655 | - | 1* | NC_000913.3 |

** complete genome assemblies already available in NCBI’s RefSeq database*

Additional file 1: Supplement Table 5

**Supplement Table 5:** Composition of the multi-species iPtgxDB (Asp-N)

| Strains | RefSeq proteins | RefSeq sProteins* | extensions to RefSeq sProteins* | additional Prodigal sProteins* | additional Chem-genome sProteins* | additional in silico sProteins* | total iPtgxDB-annotation clusters | total iPtgxDB sProtein annotation clusters* |
| --- | --- | --- | --- | --- | --- | --- | --- | --- |
| A. caccae | 3,440 | 295 | 149 | 111 | 2,409 | 80,270 | 92,405 | 83,234 |
| B. longum | 1,728 | 85 | 55 | 181 | 1,347 | 44,491 | 60,298 | 46,159 |
| B. producta | 5,682 | 577 | 346 | 319 | 3,780 | 143,796 | 168,717 | 148,818 |
| B. thetaiotaomicron | 3,067 | 384 | 205 | 99 | 1,630 | 74,732 | 82,362 | 77,050 |
| C. butyricum | 4,941 | 463 | 299 | 291 | 3,838 | 131,628 | 149,876 | 136,519 |
| C. ramosum | 3,025 | 281 | 155 | 114 | 218 | 53,727 | 58,744 | 54,495 |
| E. coli K-12 | 4,411 | 551 | 327 | 132 | 3,505 | 109,118 | 126,705 | 113,633 |
| L. plantarum | 4,148 | 391 | 153 | 192 | 284 | 70,050 | 77,200 | 71,070 |
| Combined iPtgxDB | 30,442 | 3,027 | 1,689 | 1,439 | 17,011 | 707,812 | 816,307 | 730,978 |

Additional file 1: Supplement Table 7

**Supplement Table 7:** Characteristics of reconstructed metabolic networks for SIHUMIx strains

| Species | Pathways | Reactions | Metabolites | Enzymes |
| --- | --- | --- | --- | --- |
| AC | 87 | 1617 | 1486 | 543 |
| BL | 47 | 1136 | 1004 | 292 |
| BP | 108 | 1818 | 1646 | 637 |
| BT | 98 | 1608 | 1387 | 524 |
| CB | 137 | 1917 | 1682 | 720 |
| CR | 75 | 1440 | 1340 | 487 |
| EC | 103 | 1843 | 1723 | 622 |
| LP | 94 | 1707 | 1540 | 597 |

**Supplement information 1:** Detailed description of the metaproteomics and proteomics approach taken

Cell lysis – GelFree enrichment

Bacteria cell pellets (approx. 7 x 10^9^ cells) were lysed 1 mL UPX Lysis buffer (Expedeon, USA) with 0.1% PMSF. To breakdown cell membranes 0.5 g Zirconia beads (0.1 mm) and 3 glass beads (3 mm) were added and 3 cycles of FastPrep (5.5 ms, 1 min) were performed. Afterward, samples were heated on a shaker (37°C; 10 min; 1,400 rpm). Bacterial cells were further disrupted with a sonic probe (cycle 0.5; amplitude 60%, Branson Sonifier 250, Emerson, USA), while the samples were kept on ice. Undissolved material was removed by centrifugation (10,000 × g; 10 min; 4°C). The supernatants containing the extracted protein were stored at -20°C. Protein concentration was determined with BradfordUltra (Expedeon, USA) according to kit instructions.

Cell lysis – SP3, FASP, InSolution proteolytic cleavage, C8 cartridge enrichment

The bacteria cell pellets were resuspended in 660 µL methanol. After adding 330 µL MilliQ water and 330 µL chloroform the samples were vortexed and incubated on ice for 10 min before being sonicated and centrifuged (1,700 x g; 10 min; 4°C) (see above). The interphase containing proteins was obtained and dried by evaporation of the remaining solvent in a vacuum concentrator. The pellet was resuspended in urea buffer: UT solution (8 M urea; 2 M thiourea) for In-Solution proteolytic cleavage, SP3, and C8-Cartridge enrichment or UA buffer (8 M urea in 0.1 M Tris/HCl; pH 8.5) for FASP. Protein concentration was measured with Pierce™ 660 nm Protein Assay Reagent (Thermo Fischer, USA) as per manufactures suggested.

In-Solution proteolytic cleavage

4 µg of protein in UT-solution was filled up to 20 µL with 20 mM Ammonium bicarbonate. For disulfide reduction 2 µL of 25 mM dithiothreitol- (DTT-) solution was added and the samples were heated on a thermoshaker (60°C; 1 h; 1,400 rpm). This was followed by alkylation by adding 14 µL of 20 mM ammonium bicarbonate and 4 µL of 100 mM 2-iodoacetamide and incubation at 37°C for 30 min at 1,400 rpm. Overnight enzymatic digestion was performed with trypsin or Asp-N (Promega, USA) at 37°C.

SP3

In total, 10 µg of protein lysate in UT-solution was used and 20 µL of 20 mM Ammonium bicarbonate was added. For disulfide reduction 2 µL of 25 mM Dithiothreitol- (DTT-) solution was added and the samples were heated on a thermoshaker (60°C; 1 h; 1,400 rpm). This step was followed by adding 14 µL of 20mM ammonium bicarbonate and 4 µL of 100 mM 2-iodoacetamide and incubation at 37°C for 30 min at 1,400 rpm, in the dark. SpeedBeads™ magnetic carboxylate modified particles (Sigma-Aldrich) were used for Single-pot, solid-phase-enhanced sample preparation (SP3). Beads were prepared by mixing 2 µL beads with 200 µL MilliQ water. The tubes were placed on a magnetic rack until the beads have settled to the tube wall. The supernatant was removed and beads were further rinsed with 200 µL MilliQ water twice. Protein samples were acidified by adding 5 µL 10% formic acid. 70 µL of acetonitrile was further added before transferring the mixture to the prepared beads. After 8 min incubation at RT samples were placed on a magnetic rack for 2 min and the supernatant was discarded. Beads were rinsed with 200 µL ethanol and incubated for 1 min. This step was repeated and followed by the addition of 200 µL acetonitrile, 1 min incubation and discarding the supernatant. For the proteolytic cleavage 10 µL trypsin in 100mM ammonium bicarbonate solution was added and incubated over night at 37°C. Peptides were cleaned by adding 250 µL acetonitrile to the beads, mixing and 8 min incubation off the magnetic racks. After an additional incubation for 2 min on the magnetic rack the supernatant was discarded. 180 µL of acetonitrile were added and incubated for 1 min with further discard of the supernatant. To elute peptides 20 µL MilliQ water with 2% DMSO was added. Samples were sonicated for 1 min in a waterbath and centrifuged (1,000 x g, 5 min). The supernatant was collected. After repeating the elution step the supernatants were combined and evaporated using SpeedVac. Dry peptide samples were resolved in 20 µL 0.1% formic acid.

FASP

Vivacon 500 (Sartorius, Germany) with 10 kDa MWCO membranes were equilibrated with 100 µL UA buffer and centrifuged (14,000 x g; 40 min; 20°C). All centrifugation steps were performed applying the same conditions. 60 µg protein in UA buffer was added and centrifuged. Proteins retained on the filter were incubated with 200 µL 10 mM DTT in UA buffer using a thermoshaker (37°C; 30 min; 1,400 rpm). After centrifugation, the samples were alkylated with 200 µL 50 mM IAA in UA for 10 min in the dark at RT. The samples were centrifuged and the filter membranes equilibrated with 100 µL UB (8 M urea in 0.1 M Tris/HCl; pH 8.0) followed by centrifugation. For proteolytical cleavage 10 µL UB and 80 µL trypsin or Asp-N (Promega, USA) in 80 µL 50mM ammonium bicarbonate solution was added and incubated over night at 37°C. Peptides were eluted by a centrifugation step followed by adding an additional 50 µL of 500mM NaCl solution and repeating the centrifugation. The enzymatic reaction was stopped by adding 10 µL of 10% formic acid.

C8 cartridge enrichment

Bond Elute C8 cartridges (100 mg, Agilent, USA) were equilibrated with 1 mL methanol followed by 2 mL 0.1% trifluoroacetic acid (TFA) before loading of 1 mL protein samples in UT-buffer. Afterwards, two washing steps with 1 mL 0.1% TFA were performed before eluting in two steps with 0.1% TFA:acetonitrile (3:1 v/v) and (1:1 v/v). After evaporating eluted proteins using SpeedVac they were proteolytically cleaved as described above (In-Solution proteolytic cleavage).

Peptide desalting

Extracted peptides were purified by SOLAµ (Thermo Scientific, USA) as per the manufacturer’s recommendation. After evaporation peptides were resuspended in 20 µL 0.1% formic acid.

Method assignment

The first screening for sProteins was performed using SIHUMIx bioreactor samples and all proteomic methods described above followed by trypsin proteolytic cleavage. A second screen was performed with bioreactor samples and the most promising sProtein enrichment methods: FASP, GelFree- and C8 cartridge enrichment using Asp-N as proteolytic enzyme. The third screen for sProteins was conducted on SIHUMIx single strain level. Bacteria species with promising novel sProteins, namely *Anaerostipes caccae*, *Bacteroides thetaiotaomicron*, *Blautia producta* and *Clostridium ramosum* were processed with FASP, GelFree- and C8 cartridge enrichment before proteolytic cleavage with trypsin.

**Supplement information 2:** Description of Mass spectrometric analysis

For each LC-MS/MS run 5 µL of total peptide lysates were injected into nanoHPLC (UltiMate 3000 RSLCnano, Dionex, Thermo Fisher Scientific). Peptides were trapped on a C18-reverse phase trapping column (C18 PepMap100, 300 µm x 5 mm, particle size 3 µm, Thermo Fischer Scientific, or µPACᵀᴹ Trapping column, Pharmafluidics, Belgium), followed by separation on a C18-reverse phase analytical column (Acclaim PepMap^®^ 100, 75 µm x 25 cm, particle size 3 µm, nanoViper, Thermo Fischer Scientific, or 50 cm µPACᵀᴹ column, Pharmafluidics). For separation a two-step gradient was applied (90 min 4% solvent B to 30% B, followed by 30 min from 30% B to 55% B; solvent A: 0.1% formic acid; solvent B: 80% acetonitrile, 0.1% formic acid) at 300 nL/min flowrate and a column temperature of 35°C. Mass spectrometric analysis of eluted peptides was performed on a Q Exactive HF mass spectrometer (Thermo Fisher Scientific, Waltham, MA, USA) coupled with a TriVersa NanoMate (Advion, UK) source in LC chip coupling mode. A data dependent MS/MS measurement was performed in positive mode with following settings: Full MS resolution 120 000, Full MS automatic gain control (AGC) target 3x10^6^ ions, maximum injection time for Full MS 80 ms, scan range: 350-1550 m/z, dynamic exclusion 30 s, TopN = 20, isolation window 1.6 m/z, MS/MS resolution 15 000, MS/MS AGC target 2x10^5^ ions, maximum injection time for MS/MS 120 ms; High collision dissociation energy: 28.

**Supplement information 3:** Description of Synthetic peptide analysis

To validate the peptide spectrum matches of identified novel sProteins, synthetic peptides were ordered from Thermo Fisher Scientific, USA. The synthetic peptides were resolved in 1 mL 40% acetonitril, 1% formic acid and further diluted to 1 ng/µL. MS/MS spectra was generated by direct infusion with a TriVersa NanoMate (Advion, UK) source in infusion mode. Following MS/MS settings were applied: Polarity: positive, Full MS resolution 120 000, Full MS automatic gain control (AGC) target 3x10^6^ ions, maximum injection time for Full MS 80 ms, scan range: 350-1550 m/z, no dynamic exclusion, TopN = 20, isolation window 1.6 m/z, MS/MS resolution 15 000, MS/MS AGC target 2x10^5^ ions, maximum injection time for MS/MS 120 ms; High collision dissociation energy: 28.

The matched peptide spectra were compared to synthetic peptide spectra using NIST MS Search Program v.2.0g with ±0.1 m/z precursor and product ion tolerance.

**References**

1 Rueden, C. T. *et al.* ImageJ2: ImageJ for the next generation of scientific image data. *BMC Bioinformatics* **18**, 529, doi:10.1186/s12859-017-1934-z (2017).

2 Schindelin, J. *et al.* Fiji: an open-source platform for biological-image analysis. *Nature Methods* **9**, 676-682, doi:10.1038/nmeth.2019 (2012).
